# PRSS2 remodels the tumor microenvironment via repression of Tsp1 to stimulate tumor growth and progression

**DOI:** 10.1038/s41467-022-35649-9

**Published:** 2022-12-27

**Authors:** Lufei Sui, Suming Wang, Debolina Ganguly, Tyler P. El Rayes, Cecilie Askeland, Astrid Børretzen, Danielle Sim, Ole Johan Halvorsen, Gøril Knutsvik, Jarle Arnes, Sura Aziz, Svein Haukaas, William D. Foulkes, Diane R. Bielenberg, Arturas Ziemys, Vivek Mittal, Rolf A. Brekken, Lars A. Akslen, Randolph S. Watnick

**Affiliations:** 1 Vascular Biology Program, Boston Children’s Hospital, Boston, MA USA; 2 Department of Surgery, Harvard Medical School, Boston, MA USA; 3 Hamon Center for Therapeutic Oncology Research and Department of Surgery, UT Southwestern, Dallas, TX USA; 4 Department of Cardiothoracic Surgery, Weill Cornell Medical College, New York, NY USA; 5 Department of Cell and Developmental Biology, Weill Cornell Medical College, New York, NY USA; 6 Neuberger Berman Lung Cancer Center, Weill Cornell Medical College, New York, NY USA; 7 Centre for Cancer Biomarkers CCBIO, Department of Clinical Medicine, University of Bergen, Bergen, Norway; 8 Department of Pathology, Haukeland University Hospital, Bergen, Norway; 9 Department of Urology, Haukeland University Hospital, Bergen, Norway; 10 Cancer Axis, Lady Davis Institute for Medical Research, Jewish General Hospital, Montreal, QC Canada; 11 Department of Human Genetics, McGill University, Montreal, QC Canada; 12 The program in Mathematics in Medicine, Houston Methodist Research Institute, Houston, TX USA; 13 Present Address: Vigeo Therapeutics, 86A Sherman Street, Cambridge, 02140 MA USA

**Keywords:** Cancer microenvironment, Tumour immunology

## Abstract

The progression of cancer from localized to metastatic disease is the primary cause of morbidity and mortality. The interplay between the tumor and its microenvironment is the key driver in this process of tumor progression. In order for tumors to progress and metastasize they must reprogram the cells that make up the microenvironment to promote tumor growth and suppress endogenous defense systems, such as the immune and inflammatory response. We have previously demonstrated that stimulation of Tsp-1 in the tumor microenvironment (TME) potently inhibits tumor growth and progression. Here, we identify a novel tumor-mediated mechanism that represses the expression of Tsp-1 in the TME via secretion of the serine protease PRSS2. We demonstrate that PRSS2 represses Tsp-1, not via its enzymatic activity, but by binding to low-density lipoprotein receptor-related protein 1 (LRP1). These findings describe a hitherto undescribed activity for PRSS2 through binding to LRP1 and represent a potential therapeutic strategy to treat cancer by blocking the PRSS2-mediated repression of Tsp-1. Based on the ability of PRSS2 to reprogram the tumor microenvironment, this discovery could lead to the development of therapeutic agents that are indication agnostic.

## Abstract (web summary)

Tsp-1 in the tumor microenvironment is known to suppress tumor growth and progression. Here the authors show that PRSS2 represses Tsp-1 by binding to lipoprotein receptor-related protein 1 and suggest targeting PRSS2 mediated Tsp-1 repression as a potential therapeutic strategy.

## Introduction

The paracrine, juxtacrine, and exocrine signaling between tumor cells and the non-transformed cells that constitute the tumor microenvironment (TME) are among the key drivers of tumor progression and metastasis. These intercellular signaling pathways regulate such crucial processes as tumor cell invasion and migration, angiogenesis, and immune and inflammatory cell infiltration^1–3^. Thus, the ability of a tumor to alter the activity of the cells in the microenvironment is critical for growth at the primary and metastatic sites^4–8^. Like many intracellular processes, the balance between extracellular tumor-promoting factors and tumor-inhibitory factors ultimately determines whether tumors grow and expand beyond the primary site or remain localized or even dormant. For example, pro-invasive proteases can be counteracted by protease inhibitors. Similarly, pro-angiogenic factors, such as VEGF, are balanced by anti-angiogenic factors, such as endostatin and thrombospondin-1 (Tsp-1).

Interestingly, tumor-secreted molecules often promote, or inhibit, tumor growth via more than one mechanism. For example, VEGF was identified as a pro-angiogenic factor^9,10^, but has subsequently been demonstrated to also be a potent immunosuppressive factor^11–14^. Tsp-1, on the other hand, is a potent inhibitor of angiogenesis, but also mediates the resolution of inflammation by promoting M1 polarization of macrophages^15^. We have previously identified a tumor-secreted protein, prosaposin, (PSAP) that functions as a paracrine inhibitor of primary and metastatic tumor growth^5^. PSAP inhibits tumor growth and metastasis primarily via the stimulation of the expression of Tsp-1 in myeloid-derived suppressor cells (MDSCs)^4,16^.

In the studies that identified prosaposin as a stimulator of Tsp-1, we also observed the ability of highly metastatic tumor cells to repress Tsp-1 in the TME^5^. Here, we report the use of a proteomic screen to identify the tumor-secreted, metastasis-promoting, repressor of Tsp-1 as the serine protease PRSS2. PRSS2 is alternatively known as Trypsin-2 and tumor-associated trypsin, and its overexpression has been linked to, and can induce, pancreatitis^17–19^. Further, we demonstrate that PRSS2 represses Tsp-1, not via its protease activity, but by binding to low-density lipoprotein receptor-related protein 1 (LRP1). Strikingly, the knockdown of PRSS2 in tumor cells and knockout of LRP1 in myeloid cells potently inhibited tumor growth in mouse models of breast and pancreatic cancer, with tumors having higher levels of Tsp-1 in the TME. These findings establish a previously undescribed intercellular signaling pathway that stimulates tumor growth and progression via the repression of Tsp-1. Moreover, we have demonstrated that stimulation of Tsp-1 via ectopic expression of PSAP and systemic delivery of a therapeutic peptide derived from PSAP potently inhibits primary and metastatic tumor growth in multiple tumor models^4,5,16^. Significantly, the anti-tumor therapeutic strategy of augmenting Tsp-1 expression by the PSAP peptide has also been validated in clinical trials^20,21^. Thus, based on the paracrine mechanism of the anti-tumor activity of Tsp-1, a therapeutic strategy that disrupts its repression by PRSS2 should be indication agnostic.

## Results

### Metastatic tumors repress Tsp-1 in the tumor microenvironment

**Fig. 1:**
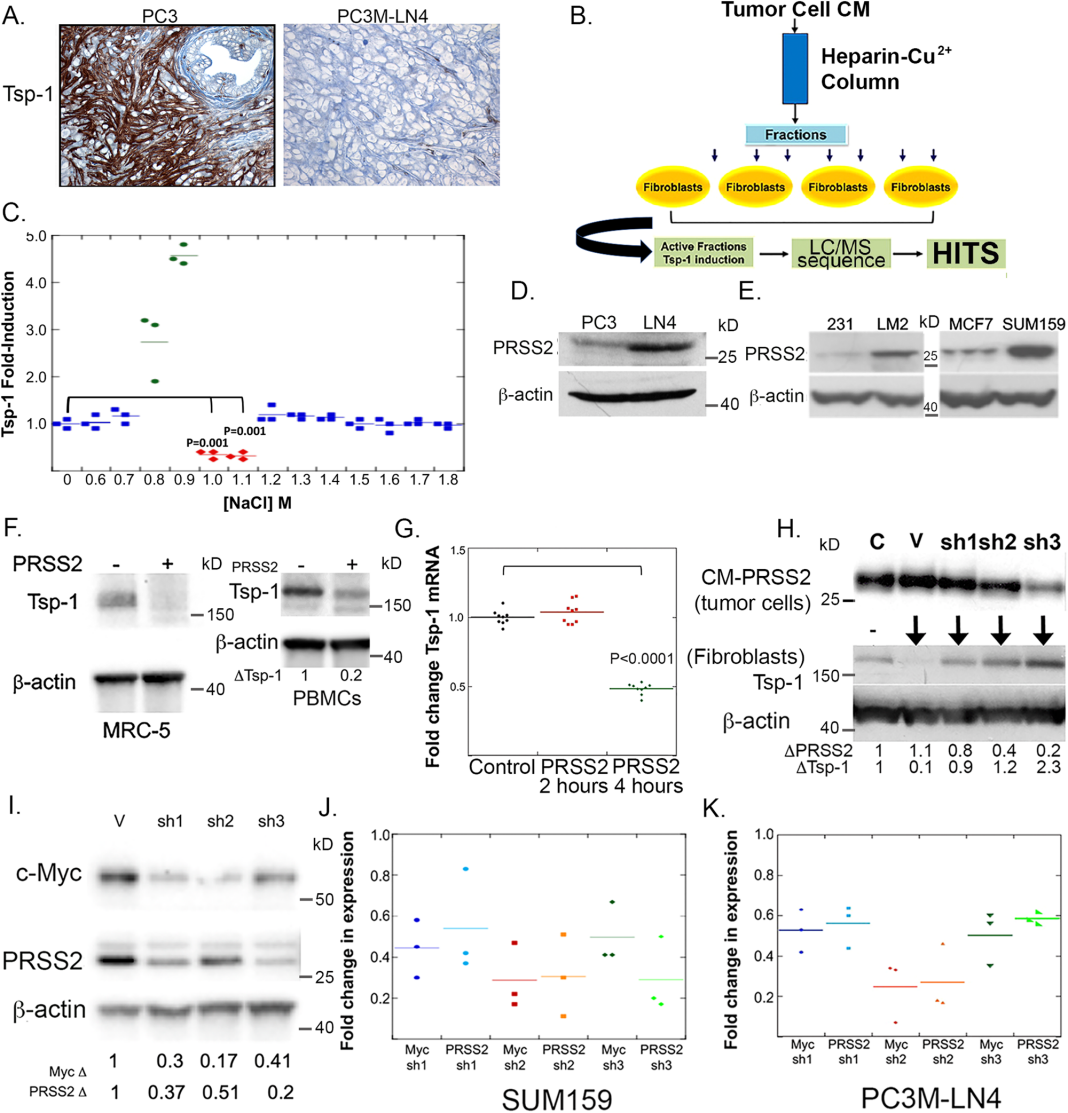
Breast and prostate cancer cells repress Tsp-1 in a paracrine manner via PRSS2. **A** Immunohistochemistry (IHC) of thrombospondin-1 (Tsp-1) expression in tumors formed in the prostate gland of SCID mice by PC3 and PC3M-LN4 prostate cancer cells (scale bar = 100 μm) (*n* = 8 per group). **B** Schematic diagram of the proteomic screening and identification of Tsp-1 repressing protein in PC3M-LN4 conditioned media. **C** ELISA of Tsp-1 expression (normalized to total protein levels) in PC3M-LN4 fractions eluted from a Heparin-sepharose-Cu^2+^ column (*n* = 3). **D** Western blot of PRSS2 and actin protein levels in PC3 and PC3M-LN4 prostate cancer cells (*n* = 3). **E** Western blot of PRSS2 and actin protein levels in MDA-MB-231 (231), MDA-MB-231-LM2 (LM2), MCF7 and SUM159 breast cancer cells (*n* = 3). **F** Tsp-1 and actin levels in MRC5 fibroblasts and primary human peripheral blood mononuclear cells (PBMCs) that were untreated (–) or treated with recombinant human PRSS2 ( + ) (*n* = 3). **G** Plot of Tsp-1 mRNA fold change, measured by real-time quantitative RT-PCR in WI38 cells treated with conditioned media of 293T cells engineered to overexpress PRSS2 (*n* = 3; *P* value calculated via two-sided Student’s *t* test). **H** Upper panel: Western blot of PRSS2 in SUM159 cells that were untransfected (C), transfected with empty pLKO.1 vector (V), or pLKO.1 vector expressing three independent shRNA sequences directed against PRSS2 (sh1, sh2, sh3). Lower panel: Western blot of Tsp-1 and actin in WI38 cells that were untreated (–) or treated with conditioned media from SUM159 cells with empty vector (V) or PRSS2 shRNA (*n* = 3). **I** Western blot of Myc, PRSS2 and actin in SUM159 cells transfected with vector control (V) or three shRNA sequences directed against c-Myc (sh1, sh2 and sh3) (*n* = 3). **J** Dot plot of fold change of Myc and PRSS2 in SUM159 cells transfected with vector control (V) or three shRNA sequences directed against c-Myc (sh1, sh2, and sh3 (*n* = 3). **K** Dot plot of fold change of Myc and PRSS2 in PC3M-LN4 cells transfected with vector control (V) or three shRNA sequences directed against c-Myc (sh1, sh2, and sh3) (*n* = 3).

We have previously reported that highly metastatic human breast and prostate tumor cells derived from weakly metastatic cell lines via serial in vivo passaging repress the expression of the anti-tumorigenic protein Tsp-1 in the TME^5^. Staining of weakly metastatic PC3 prostate tumor xenografts and highly metastatic PC3M-LN4 xenografts confirmed that Tsp-1 was highly expressed in the TME of PC3 but not PC3M-LN4 xenografts (Fig. 1A).

To identify the tumor-secreted protein responsible for the paracrine repression of Tsp-1 we utilized a proteomic screening method previously used to identify prosaposin as a stimulator of Tsp-1 expression (Fig. 1B)^5^. Briefly, CM was fractionated over a heparin-sepharose/Cu^2+^ column and proteins were eluted with a linear gradient of NaCl plus 10 mM imidazole. Collected fractions were dialyzed into PBS and used to treat primary human lung fibroblasts and Tsp-1 expression was analyzed by ELISA and western blot. We found that the Tsp-1 repressing activity was present in fractions that eluted with 1.0 and 1.1 M NaCl (Fig. 1C and Supplementary Fig. S1). These two fractions, along with the inactive fractions that eluted at 0.9 and 1.2 M NaCl, were concentrated and analyzed by tandem liquid chromatography-mass spectrometry (LC-MS) analysis (Supplementary Fig. S2). Analysis of the LC-MS results yielded only one protein that was present in both of the Tsp-1 repressing fractions and absent in the inactive fractions, the serine protease PRSS2. PRSS2 is an anionic trypsinogen and is also referred to as tumor-associated trypsin (TAT). PRSS2 has been found at elevated levels in tissue and serum of gastric, pancreatic, prostate, and ovarian cancer patients^22–26^.

We validated expression of PRSS2 in PC3M-LN4 cells by western blot and found that PC3M-LN4 cells expressed ~tenfold higher levels of PRSS2 than the weakly metastatic PC3 cells, which stimulate Tsp-1 expression in lung fibroblasts^5^ (Fig. 1D). We then analyzed a set of human breast cancer cell lines for PRSS2 expression and found that its expression level correlated with the metastatic potential of the cell lines (Fig. 1E). Specifically, MDA-LM2, a metastatic derivative of MDA-MB-231 expressed significantly higher levels of PRSS2 than its parental cell line. Additionally, SUM159, a metastatic triple-negative breast cancer cell line expresses significantly higher levels of PRSS2 than the ER + and non-metastatic cell line MCF7.

### PRSS2 is necessary and sufficient for paracrine repression of Tsp-1

Having identified PRSS2 as being present in Tsp-1 repressing fractions of PC3M-LN4 cells, we sought to validate that it represses Tsp-1. We treated primary human lung fibroblasts with purified recombinant human PRSS2 (rhPRSS2) and found that rhPRSS2 was sufficient to repress Tsp-1 expression in WI38 and MRC5 fibroblasts (Fig. 1F and Supplementary Fig. S3). We previously demonstrated that prosaposin stimulated Tsp-1 in Cd11b+/Gr1+ MDSCs and that these cells expressed higher levels of Tsp-1 than other types of PBMCs^4,16^. Accordingly, we also treated primary human PBMCs with PRSS2 and found, by western blot, and observed an average of 73% reduction in Tsp-1 protein levels in these cells (0.27+/– 0.033; *P* = 0.002 by Student’s *t* test) (Fig. 1F). We then sought to determine whether Tsp-1 was regulated at the mRNA or protein level by the paracrine signaling of PRSS2. To that end, we measured Tsp-1 mRNA levels by real-time quantitative reverse transcription PCR (qRT-PCR). We found that Tsp-1 mRNA levels were decreased by 51% in fibroblasts treated with conditioned media of 293 T cells engineered to ectopically express PRSS2 (0.485+/– 0.01; *t* < 0.0001 by Student’s *t* test) (Fig. 1G).

To determine if PRSS2 was required for the repression of Tsp-1 we silenced its expression in the highly metastatic breast cancer cell line SUM159 via lentiviral transduction of shRNA specific for PRSS2 (Fig. 1H). Consistent with the proposed activity of PRSS2, we found that the level of Tsp-1 induction in target cells correlated with the level of repression of PRSS2 with three independent shRNA constructs transduced into SUM159 cells (Fig. 1H). These findings indicate that PRSS2 is necessary and sufficient for the repression of Tsp-1 in human lung fibroblasts.

We previously demonstrated that the expression of Tsp-1 and prosaposin is repressed in a c-Myc-dependent manner in highly metastatic cells PC3M-LN4 (derived from PC3 cells) and MDA-LM2 (derived from MDA-MB-231 cells)^5^. Due to the observation that these metastatic cell lines repressed prosaposin concomitantly with the upregulation of PRSS2, we examined whether PRSS2 expression was also regulated by c-Myc. shRNA-mediated silencing of c-Myc by 3 independent shRNA sequences resulted in a 46, 69, and 71% reduction of PRSS2 expression in SUM159 (Fig. 1I, J) and 44, 73 and 41% in PC3-LN4 (Fig. 1K) cells consistent with the level of c-Myc knockdown. These findings indicate that PRSS2 expression is c-Myc dependent and regulated in the opposite manner as prosaposin.

### PRSS2 enzymatic activity is not required for repression of Tsp-1

The observation that PRSS2 expression is necessary and sufficient to repress Tsp-1 in a paracrine-acting fashion, suggests two possible mechanisms. The first is that PRSS2 acts as a protease to cleave a substrate that then interacts with a cell surface receptor to repress Tsp-1. The second is that PRSS2, itself, is a ligand for a cell surface receptor and represses Tsp-1 by direct binding and activation of a signal transduction cascade culminating in the repression of Tsp-1.

**Fig. 2:**
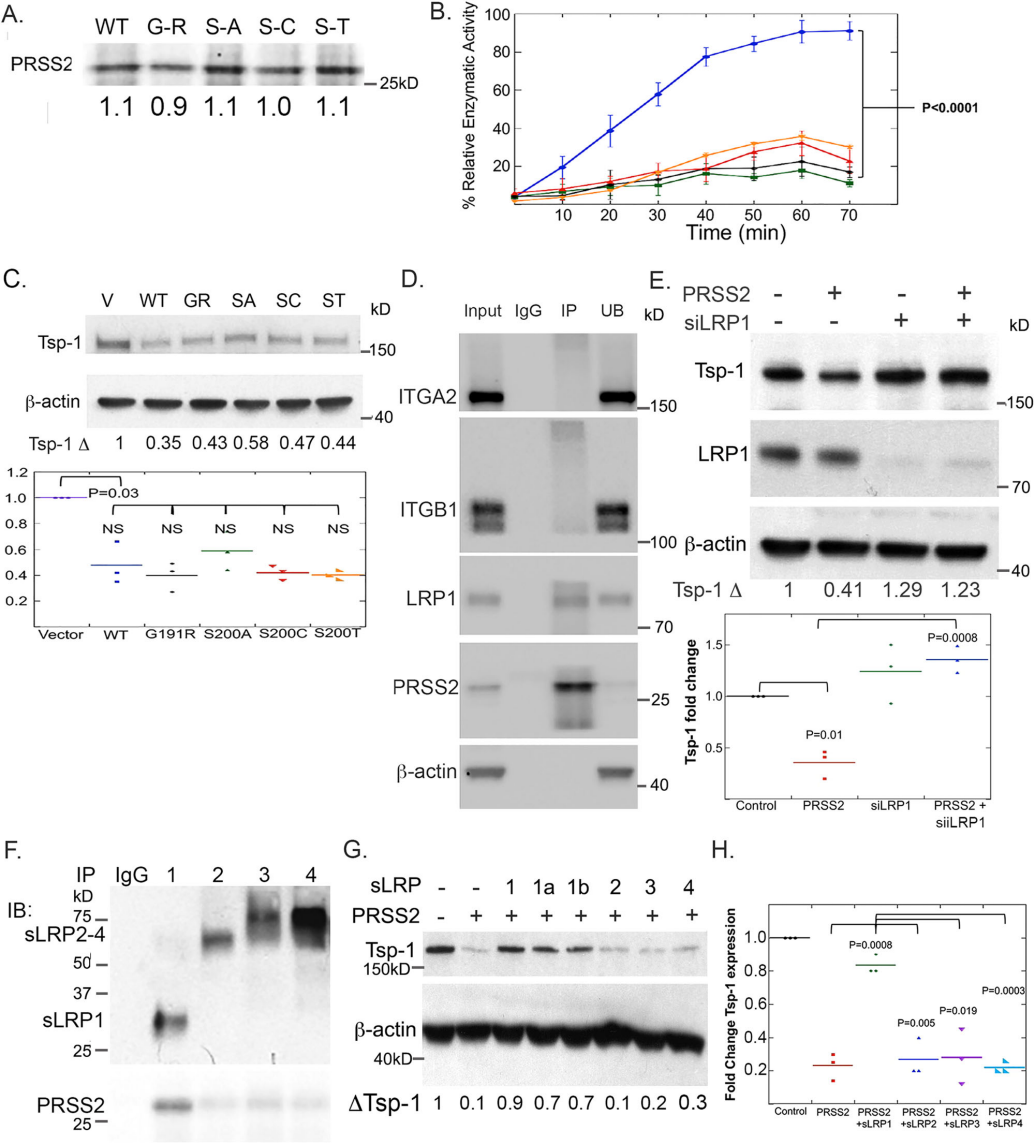
PRSS2 represses Tsp-1 via enzyme-independent binding to LRP1. **A** Western blot of PRSS2 protein levels in conditioned media of 293T cells transfected with WT PRSS2 (blue), PRSS2-G191R (black), PRSS2-S200A (green), PRSS2-S200C (red), and PRSS2-S200T (orange) (*n* = 3). **B** Plot of enzymatic activity WT and mutant PRSS2 proteins relative to WT PRSS2 (%) (*n* = 3) (*P* value calculated via two-sided Student’s *t* test; error bars depict SEM). Western blots of: **C** Tsp-1 and actin expression in WI38 fibroblasts treated with CM from 293T cells transfected with empty pCMV-Sport6 vector or (pCMV-Sport6 -PRSS2 or a mutant version of PRSS2 (GR = G191R, SA = S200A, SC = S200C, ST = S200T) (*n* = 3; *P* value calculated via two-sided Student’s *t* test). **D** Integrin α2 (ITGA2), Integrin β1 (ITGB1), LRP1, PRSS2, and actin following immunoprecipitation with control IgG, or α-PRSS2 (IP) antibody and unbound protein (UB) in WI38 cells (*n* = 3). **E** Dot plot of all replicates of Tsp-1, LRP1, and actin in WI38 cells that were untreated (–) or treated with PRSS2 (+) in the presence (+) or absence (–) of siRNA directed against LRP1 (*n* = 3; *P* value calculated via two-sided Student’s *t* test). **F** Secreted truncation mutants of LRP1 (sLRP1) containing binding domains 1–4, PRSS2, and actin from an immunoprecipitation experiment with α-myc epitope antibody (*n* = 4). **G** Tsp-1 and actin in WI38 cells that were untreated (–) or treated with PRSS2 (+) in the absence (–) or presence of secreted truncated mutants of PRSS2 comprised of binding domains 1, 1a, 1b, 2, 3, and 4 (*n* = 3). **H** Dot plot of quantitation of Tsp-1 western blots from all replicates of WI38 cells that were untreated (–) or treated with PRSS2 (+) in the absence (–) or presence of secreted truncated mutants of PRSS2 comprised of binding domains 1, 2, 3, and 4 (*n* = 3; *P* value calculated via two-sided Student’s *t* test).

To test the first hypothesis, we generated point mutations in PRSS2 that inactivate its enzymatic activity. In addition to making mutations in the active site, by mutating the serine at residue 200 to alanine, threonine, and cysteine (S200A, S200T, and S200C) we also generated a glycine to arginine substitution at residue 191 (G191R). This mutation has been identified as an inactivating mutation that confers resistance to familial pancreatitis^27^. We then ectopically expressed these four mutant versions of PRSS2 in 293T cells and used the conditioned media to treat lung fibroblasts. We found that all of the mutant proteins were expressed at levels comparable to wild-type PRSS2 protein (Fig. 2A). We then used a colorimetric assay to measure the enzymatic activity of the mutant PRSS2 proteins. The results of this assay confirmed that the G191R and S200A mutants were enzymatically inactive and S200T and S200C retained minimal enzymatic activity (Fig. 2B). Strikingly, the CM containing the PRSS2 mutants was able to repress Tsp-1 to the same relative degree as the wild-type protein (WT = 0.48; G191R = 0.40; S200A = 0.59, S200C = 0.42, S200T = 0.40; *P* = 0.53, 0.49, 0.62, and 0.51, respectively, (Fig. 2C). These findings indicate that the enzymatic activity of PRSS2 is not required for repression of Tsp-1 expression and suggest that PRSS2 may function as a ligand for a cell surface receptor.

### PRSS2 is a ligand for LRP1

An examination of the existing literature revealed no reports of PRSS2 as a ligand for a cell surface receptor. To identify potential PRSS2 interacting proteins we performed a co-immunoprecipitation experiment, in which CM from 293T cells transfected with a plasmid expressing PRSS2 was mixed with cell lysates from WI38 lung fibroblasts and PRSS2 was immunoprecipitated. The immunoprecipitates were run on an SDS-polyacrylamide gel, visualized by silver staining (Supplementary Fig. S4) and subsequently analyzed by LC/MS. Results of the LC/MS analysis identified 325 proteins, with a minimum of three peptide fragments, that were present in the immunoprecipitate of α-PRSS2 but not the IgG control (Supplementary Fig. S5). We performed gene ontogeny analysis (Panther) and independently searched the list of PRSS2-precipitated proteins for known cell surface receptors. This analysis yielded only three potential candidates: integrin α2 (ITGA2), integrin β1 (ITGB1), and low-density lipoprotein-related receptor protein 1 (LRP1).

To confirm that PRSS2 specifically binds these proteins, we repeated the immunoprecipitation experiment and performed western blot analysis for each receptor. We found that PRSS2 reproducibly co-immunoprecipitated with LRP1 but was not able to co-immunoprecipitate ITGA2 or ITGB1 (Fig. 2D). To functionally validate these findings, we silenced expression of LRP1 in primary lung fibroblasts via siRNA and confirmed knockdown by western blot (Fig. 2E). We then treated these cells with conditioned media from 293T cells that were transiently transfected with PRSS2. We found that CM containing PRSS2 repressed Tsp-1 by 58%, +/–1.3%, in mock-transfected target cells (Fig. 2E). Conversely, silencing LRP1 in WI38 cells, via siRNA, abolished the repression of Tsp-1 by PRSS2 (1.08-fold change, *P* = 0.75 by ANOVA). As a control, silencing LRP1 alone had no significant effect on Tsp-1 expression compared to mock-transfected cells (124% +/– 17%; *P* = 0.386 by Student’s *t* test). These results indicate that LRP1 is the cell surface receptor that mediates PRSS2 repression of Tsp-1.

As this is the first observation of PRSS2 binding to LRP1 we sought to identify the binding domain of LRP1 that mediates the interaction between the two proteins. For this experiment, we incubated the CM from SUM159 cells with CM from 293T cells transfected with vectors expressing soluble (secreted) truncation mutants of LRP1 consisting of the four binding domains of the protein fused to an N-terminal Myc epitope tag^28^. We found that only LRP1 binding domain 1 co-immunoprecipitated with PRSS2 (Fig. 2F). While LRP1 has been reported to have over 50 ligands^29^, PRSS2 represents the first identified protein to bind independently to LRP1 domain 1 (as opposed to proteins, such as α2-macroglobulin, which bind to domain 1 but not independently of binding to domain 2 and 4)^28–30^.

We then tested the functional significance of the interactions between PRSS2 and LRP1 by treating lung fibroblasts with CM from 293T cells ectopically expressing PRSS2 in the presence and absence of soluble versions of the LRP1 truncation proteins. The rationale behind this experiment is that if the interactions between PRSS2 and domains 1 and 3 of LRP1 are required for the repression of Tsp-1, then the soluble truncated proteins should serve as decoys to sequester PRSS2 and prevent it from binding to LRP1. Consistent with the results from the co-IP experiment, incubation of PRSS2 with binding domains 1 and 1a of LRP1 blocked the repression of Tsp-1, while incubation with binding domains 2 and 3 had no effect (Fig. 2G, H).

**Fig. 3:**
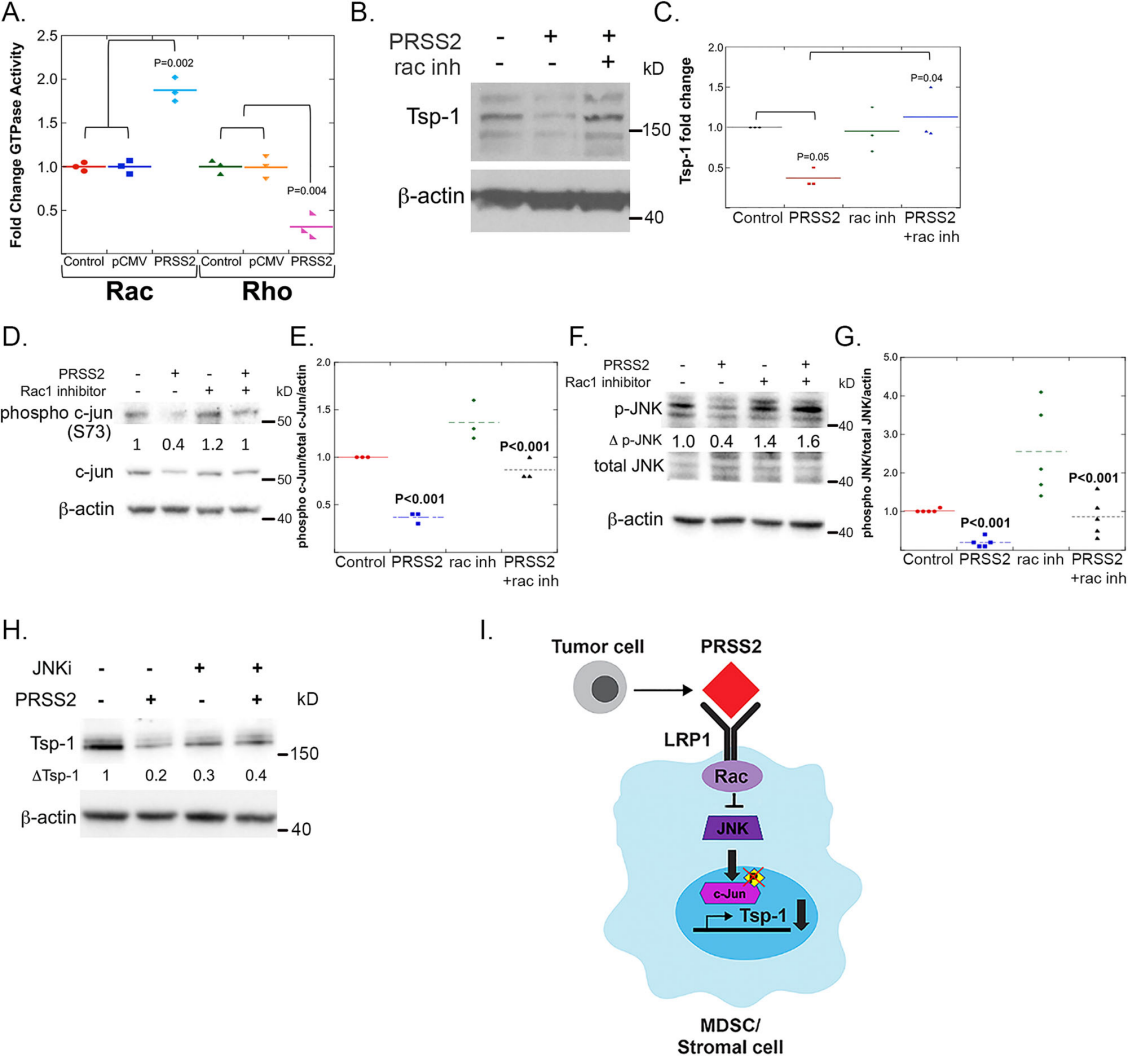
PRSS2 represses Tsp-1 by activating Rac downstream of LRP1. **A** Dot plot of GTP-bound Rac and Rho in WI38 cells that were untreated (control), treated with CM from 293T cells transfected with empty vector (pCMV) or PRSS2 (PRSS2) (*n* = 3; *P* value calculated via two-sided Student’s *t* test). **B** Western blot of Tsp-1 and actin expression in WI38 cells that were untreated (–) or treated with PRSS2 (+) in the absence (–) and presence (+) of a small molecule inhibitor of Rac1 NSC23766 (*n* = 3). **C** Dot plot of quantitation of Tsp-1 western blots from all replicates of WI38 that were untreated (–) or treated with PRSS2 (+) in the absence (–) and presence (+) of NSC23766 (*n* = 3; *P* value calculated via two-sided Student’s *t* test). **D** Western blot of phospho c-Jun, total c-Jun and actin in WI38 cells that were treated with saline (–), PRSS2, Rac1 inhibitor NSC23766, or PRSS2 and NSC23766 in combination (*n* = 3). **E** Dot plot of quantitation of phospho c-Jun, total c-Jun, and actin in WI38 cells that were treated with saline (–), PRSS2, Rac1 inhibitor NSC23766, or PRSS2 and NSC23766 in combination (*P* values calculated by two-sided ANOVA; *n* = 3). **F** Western blot of phosphor-JNK, total JNK and actin in WI38 cells that were treated with saline (–), PRSS2, Rac1 inhibitor NSC23766, or PRSS2 and NSC23766 in combination (*n* = 5). **G** Dot plot of quantitation of phospho-JNK, total JNK and actin in WI38 cells that were treated with saline (–), PRSS2, Rac1 inhibitor NSC23766, or PRSS2 and NSC23766 in combination (*P* values calculated by two-sided ANOVA; *n* = 5). **H** Western blot of Tsp-1 and actin in WI38 cells that were treated with saline (–), PRSS2, JNK1 inhibitor III, or PRSS2 and JNK1 inhibitor III in combination (*n* = 3). **I** Schematic diagram of PRSS2 repression of Tsp-1 via LRP1-Rac signaling.

Finally, we sought to determine the components of the signal transduction cascade downstream of LRP1 leading to the repression of Tsp-1. It has been demonstrated that LRP1 ligands can activate Rho and Rac GTPases^31,32^. To determine whether Rho or Rac was activated by PRSS2 binding to LRP1 we measured the activation of both GTPases using plate-based G-LISA assays. We found that CM from 293T cells ectopically expressing PRSS2 stimulated Rac-GTPase activity ~twofold in WI38 cells compared to CM from empty vector-transfected cells (+/– 0.08, *P* = 0.002 by Student’s *t* test) (Fig. 3A). Conversely, and consistent with published reports that Rac and Rho activity are reciprocally regulated^33–35^, treatment of WI38 cells with PRSS2 CM resulted in a 3.2-fold decrease in Rho-GTPase activity (+/– 0.08, *P* = 0.004 by Student’s *t* test) (Fig. 3A).

To functionally validate these findings, we treated lung fibroblasts with CM from SUM159 cells in the presence and absence of the small molecule inhibitor of Rac1, NSC23766^36^. We found that while CM from SUM159 cells repressed Tsp-1 by 2.3-fold (+/– 0.13, *P* = 0.05 by Student’s *t* test), treatment with the Rac1 inhibitor completely abrogated the repression of Tsp-1 (*P* = 0.04 by Student’s *t* test) (Fig. 3B, C). These findings demonstrate that PRSS2 represses Tsp-1 via activation of Rac1 downstream of binding to LRP1.

To determine how activation of Rac1 could result in the repression of the transcription of Tsp-1, we examined the phosphorylation of c-Jun, which has been shown to be a transcriptional activator of Tsp-1 when complexed with c-Fos to form AP-1^37^. Moreover, and consistent with this hypothesis, LRP1 has been demonstrated to inhibit JNK activity^38,39^. We found that levels of phosphorylated c-Jun were decreased by 2.7-fold in fibroblasts treated with PRSS2 (*n* = 3, 1 vs 0.36; *P* < 0.001 by ANOVA (Fig. 3D, E). We then examined the effects of PRSS2 on the activation of c-Jun N-terminal Kinase (JNK) and found that PRSS2 inhibited the phosphorylation of JNK by 3.75-fold (*n* = 5, *P* < 0.001 by ANOVA) (Fig. 3F, G). Critically, the inhibition of the phosphorylation of both c-Jun and JNK by PRSS2 was abrogated by co-treatment with the Rac1 inhibitor, NSC23766 (*P* < 0.001 and 0.005, respectively) (Fig. 3F, G). Finally, we demonstrate that treatment of WI38 cells with JNK inhibitor results in a 2.5-fold decrease in Tsp-1 expression in fibroblasts (1 vs 0.4, *n* = 4, *P* < 0.001 by ANOVA) (Fig. 3H). Consistent with these results, Rac1 activation has been shown to inhibit c-Jun phosphorylation and JNK activation^40,41^. Moreover, treatment of cells with PRSS2 and JNK inhibitor did not result in further repression of Tsp-1 compared to PRSS2 alone or JNK inhibitor alone, suggesting that PRSS2 and JNK are in the same pathway (Fig. 3I). Taken together, these findings indicate that PRSS2 binds to LRP1 and activates Rac1, which inhibits JNK activation decreasing the levels of phosphorylated c-Jun resulting in decreased transcription of Tsp-1.

Since LRP1 is also an endocytic receptor, we sought to determine whether PRSS2 was taken up by fibroblasts following treatment with CM from PRSS2-overexpressing 293T cells. We found that wild-type fibroblasts had increased levels of PRSS2 within one hour of treatment, but cells silenced for LRP1 did not (Supplementary Fig. S6). These findings suggest that in addition to inducing a Rac-mediated signal transduction cascade leading to the repression of Tsp-1, PRSS2 is also endocytosed by LRP1.

### PRSS2 levels correlate with aggressive features of breast and prostate carcinoma

**Fig. 4:**
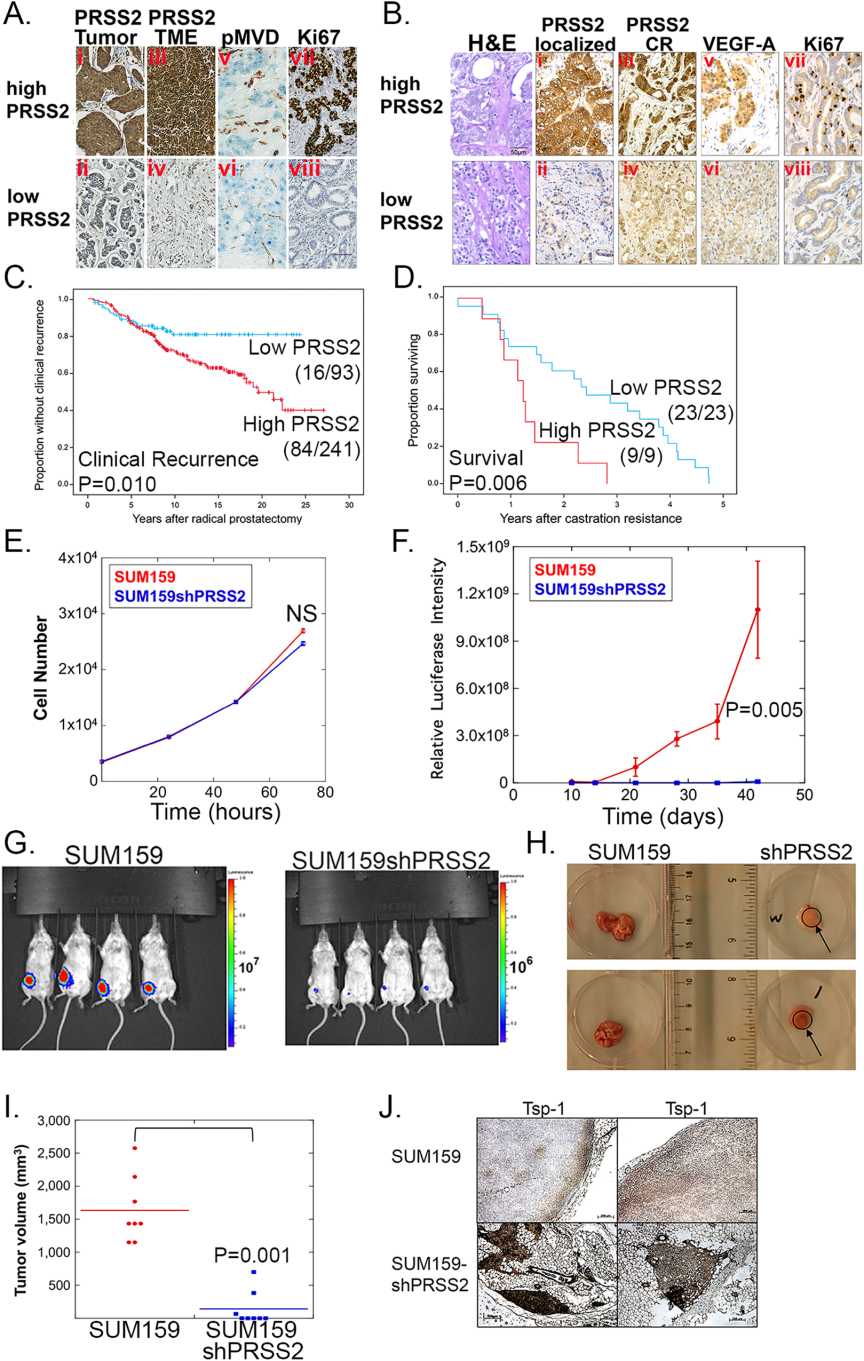
PRSS2 is required for efficient tumor formation of SUM159 cells. **A** Immunohistochemical analysis of breast cancer patient Series 1 (*n* = 544 patients) for expression of: (i) PRSS2 in tumors with strong (high) PRSS2 expression in tumor cells, (ii) PRSS2 in tumors with weak (low) PRSS2 expression in tumor cells, (iii) PRSS2 in tumors with strong PRSS2 expression in TME, (iv) PRSS2 in tumors with weak PRSS2 expression in the TME; (v) proliferating microvessel density (pMVD) in tumors with strong PRSS2 expression, (vi) pMVD in tumors with weak PRSS2 expression; (vii) proliferation (Ki67) in tumors with strong PRSS2 expression; and (viii) Ki67 in tumors with weak PRSS2 expression (scale bar = 50 μm, original magnification ×400). **B** H&E staining and immunohistochemical analysis of prostate cancer patient series (*n* = 458 patients) for expression of: (i) PRSS2 in tumors with strong (high) tumor cell expression of PRSS2 in localized prostatic carcinoma; (ii): PRSS2 in tumors with weak (low) tumor cell expression of PRSS2 in localized prostatic carcinoma; (iii) PRSS2 in tumors with strong expression of PRSS2 in castration-resistant carcinoma (CR); (iv) PRSS2 in tumors with weak expression of PRSS2 in castration-resistant carcinoma; (v) VEGF-A in localized carcinoma with strong PRSS2 expression; (vi) VEGF-A in localized carcinoma with weak PRSS2 expression; (vii) Ki67 in localized carcinoma with strong PRSS2 expression; (viii) Ki67 in localized carcinoma with weak PRSS2 expression (scale bar = 50 μm, original magnification ×400). **C** Kaplan–Meier curve of clinical progression following radical prostatectomy of prostate cancer patients with strong and weak expression of PRSS2 (*P* values were determined via two-sided log-rank test). **D** Kaplan–Meier curve of overall survival following the acquisition of castration resistance of prostate cancer patients with strong and weak expression of PRSS2 (*P* values were determined via a two-sided log-rank test). **E** Plot of in vitro proliferation of SUM159 and SUM159shPRSS2 cells over 3 days (no significant difference was determined via two-sided ANOVA). **F** Plot of in vivo luciferase activity of orthotopic tumors formed by mammary gland injection of SUM159 and SUM159shPRSS2 cells (*P* value was determined via two-sided Mann–Whitney *U* test; error bars depict SEM). **G** In vivo luciferase imaging of mice bearing SUM159 (upper panel) and SUM159shPRSS2 (lower panel) tumors. **H** Photographs of tumors formed by SUM159 and SUM159shPRSS2 cells. **I** Plot of the volume of tumors formed by SUM159 and SUM159shPRSS2 cells (*P* value calculated via two-sided Mann–Whitney *U* test). **J** Immunohistochemistry of Tsp-1 in tumors formed by SUM159 and SUM159shPRSS2 cells (*n* = 8 mice per group) (scale bar = 200 μm).

To determine whether PRSS2 has clinical relevance as a potential therapeutic target, we evaluated its expression by immunohistochemistry in series of human breast and prostate cancers (Fig. 4A, B and Supplementary Fig. S7). In a population-based breast cancer series (Series 1, *n* = 518), strong and consistent associations were found between PRSS2 expression and multiple features of aggressive tumors, such as high histologic grade, lack of expression of estrogen receptor (ER), tumor cell proliferation (by Ki67 expression), CK5/6 expression (basal marker), and increased angiogenesis (by pMVD and GMP) (Table 1). In a *BRCA*-based breast cancer series (Series 2, *n* = 202), which was enriched for cases with germline *BRCA1* or *BRCA2* mutations, strong associations with high histologic grade, tumor cell proliferation (by mitotic count), and p53 expression were present (Supplementary Table 1). In contrast, no associations with *BRCA*-status were found. Notably, these associations (Series 1–2) were found for PRSS2 levels in both tumor epithelium and when recorded separately in the stromal compartment. PRSS2 levels in tumor cells and in the associated stroma was significantly associated (Fig. 4A) (Spearman’s correlation: Series 1, rho = 0.36, *P* < 0.0005; Series 2, rho = 0.45, *P* < 0.0005).

**Table 1:** Breast cancer (Series 1)

Variables	PRSS2 epithelium					PRSS2 TME				
	Low (*n* = 458) *n* %	High (*n* = 60) *n* %	OR	95% CI	*P* value^a^	Low (*n* = 428) *n* %	High (*n* = 90) *n* %	OR	95% CI	*P* value^a^
**Grade**					<0.001					<0.001
1–2	391 (91.1)	38 (8.9)	1.0			369 (86.0)	60 (14.0)	1.0		
3	67 (75.3)	22 (24.7)	3.4	1.9, 6.1		59 (66.3)	30 (33.7)	3.1	1.9, 6.1	
**ER**					0.003					0.001
Pos	396 (90.2)	43 (9.8)	1.0			373 (85.0)	66 (15.0)			
Neg	62 (78.5)	17 (21.5)	2.5	1.4, 4.7		55 (69.6)	24 (30.4)	2.5	1.4, 4.3	
**Mitotic ct**^b^					<0.001					<0.001
Low ≤5.5	349 (91.8)	31 (8.2)	1.0			328 (86.3)	52 (13.7)			
High >5.5	103 (78.6)	28 (21.4)	3.1	1.8, 5.3		93 (71.0)	38 (29.0)	2.6	1.6, 4.2	
**Ki67**^b^					<0.001					<0.001
Low ≤31.5	351 (91.6)	32 (8.4)	1.0			333 (86.9)	50 (13.1)			
High >31.5	101 (78.9)	27 (21.1)	2.9	1.7, 5.1		88 (68.8)	40 (31.2)	3.0	1.9, 4.9	
**CK5/6**^c^					0.008					<0.001
Neg = 0	401 (98.9)	45 (10.1)	1.0			378 (84.8)	68 (15.2)			
Pos >0	52 (78.8)	14 (21.2)	2.4	1.2, 4.7		44 (66.7)	22 (33.3)	2.4	1.0, 6.0	
**pMVD**^d^					0.013					0.004
Low <4.59	112 (90.3)	12 (9.7)	1.0			109 (87.9)	15 (12.1)			
High ≥4.59	34 (75.6)	11 (24.4)	3.02	1.2, 7.5		31 (68.9)	14 (31.1)	3.3	1.4, 7.5	
**GMP**^d^					0.006					0.005
Absent	116 (89.9)	13 (10.1)	1.0			112 (86.8)	17 (13,2)			
Present	29 (72.5)	11 (27.5)	3.4	1.4, 8.3		27 (67.5)	13 (32.5)	3.2	1.4, 7.3	

*n* number of patients, *OR* odds ratio, *CI* confidence interval, *ER* estrogen receptor, *CK5/6* cytokeratin 5/6, *pMVD* proliferative microvessel density, *GMP* glomeruloid microvascular proliferation.

Series 1 (*n* = 518).

^a^Pearson’s chi-squared test (two-sided).

^b^Cutoff value by upper quartile. Seven cases lack information on Ki67 and mitotic count (mitoses/mm^2^).

^c^Six cases lack information on CK5/6 status.

^d^Three hundred and forty-nine cases lack information on pMVD (Nestin + /Ki67+ vessels) and GMP status.

In localized prostate cancer (Series 1, *n* = 338), PRSS2 expression in tumor cells was associated with increased tumor cell proliferation (by Ki67 expression), and with increased VEGF-A (Fig. 4B and Table 2). By univariate survival analyses (Series 1; *n* = 338), strong PRSS2 was associated with a shorter time to clinical recurrence (*P* = 0.010), and it was borderline associated with biochemical recurrence (*P* = 0.071) (Fig. 4C). By multivariate survival analyses (Series 1, *n* = 338), where PRSS2 was included in addition to the standard prognostic variables Gleason score (≥4 + 3 versus ≤3 + 4), pathological stage (≥pT3 versus pT2) and preoperative s-PSA (dichotomized by upper quartile), strong PRSS2 independently predicted biochemical recurrence, loco-regional recurrence, and clinical recurrence (HR = 1.4–2.5, *P* = 0.001–0.052), together with Gleason score and pathological stage, and for biochemical recurrence, s-PSA (Supplementary Table 2).

**Table 2:** Associations between PRSS2 expression and selected biomarkers in localized prostatic carcinomas and castration-resistant prostate cancer

Variables		PRSS2^a^		
		Low *n* (%)	High *n* (%)	*P* value^b^
**Localized prostate cancer**				
**Ki67**^c^				0.051
Low		18 (36)	32 (64)	
High		10 (19)	43 (81)	
**VEGF-A**^d^				0.009
Low		27 (33)	54 (67)	
High		1 (5)	20 (95)	
**PSAP**^e^				<0.0005
High		28 (36)	49 (64)	
Low		0 (0)	25 (100)	
**CD8**^e^				0.091
High		64 (26)	186 (74)	
Low		27 (36)	49 (64)	
**FoxP3**^e^				0.030
Low		15 (44)	19 (56)	
High		76 (26)	212 (74)	
**Castration-resistant prostate cancer**				
**CD8**^e^				<0.0005
High	23 (89)		3 (11)	
Low	0 (0)		6 (100)	
**FoxP3**^e^				0.654
Low	5 (63)		3 (37)	
High	18 (75)		6 (25)	

^a^Cytoplasmic expression, cutoff by median (localized prostate cancer) or upper quartile (CRPC).

^b^Pearson Chi-square test or Fisher’s exact test (two-sided).

^c^Cutoff by median.

^d^Cutoff by upper quartile.

^e^Cutoff by lower quartile.

Strikingly, and consistent with the observation that c-Myc oppositely regulates PRSS2 and PSAP, PRSS2 was strongly and inversely associated with PSAP expression (Table 2). Specifically, of the 25 patients with low levels of PSAP, all had high levels of PRSS2. Finally, by univariate survival analyses of castration resistance prostate cancer (*n* = 32), strong PRSS2 expression was associated with a shorter time from diagnosis to death (*P* = 0.006) (Fig. 4D).

### PRSS2 is required for efficient tumor growth

The observations that more aggressive breast and prostate cancer cell lines express higher levels of PRSS2 than their less metastatic counterparts coupled with the corroboration of these findings in patient samples, led us to hypothesize that disrupting PRSS2 expression could inhibit tumor growth. To test this hypothesis, we silenced PRSS2 in SUM159 cells via shRNA and measured the proliferation rates of vector control and shPRSS2 cells. We found that vector control and SUM159shPRSS2 cells had no statistically significant difference in in vitro cellular proliferation (Fig. 4E). We then injected 1 × 10^6^ vector control and SUM159shPRSS2 cells, expressing firefly luciferase, orthotopically into the mammary gland of SCID mice (*n* = 8 mice per cohort). After 40 days, the vector control tumors had reached a significant size, as determined by luciferase intensity and gross visual inspection (Fig. 4F–I). Conversely, the SUM159shPRSS2 cells formed tumors that were barely detectable by luciferase activity and undetectable by gross visual inspection (Fig. 4F–I). In fact, 5/8 mice injected with SUM159shPRSS2 cells developed tumors smaller than 4 mm^3^ (less than 2 mm in diameter) and on average the tumors were 11.5-fold smaller than tumors formed by SUM159 vector control cells (1657 mm^3^ vs 144 mm^3^, *P* < 0.001 by Mann–Whitney *U* test) (Fig. 4F, I).

We then examined the tumors histologically by H&E and IHC for Tsp-1 expression. We found that Tsp-1 expression was significantly higher in tumors formed by the shPRSS2 cells than the vector control cells, where it was almost undetectable (Fig. 4J). Of note, the size of the shPRSS2 tumors (1–2 mm in diameter) was consistent with previously published reports of dormant tumors in which Tsp-1 was highly expressed, either endogenously or ectopically^42,43^. These findings suggest that tumor-secreted PRSS2 stimulates tumor growth via paracrine signaling to repress Tsp-1 in the tumor microenvironment and not via tumor cell-autonomous effects.

### Loss of PRSS2 does not inhibit tumor growth in the absence of Tsp-1

**Fig. 5:**
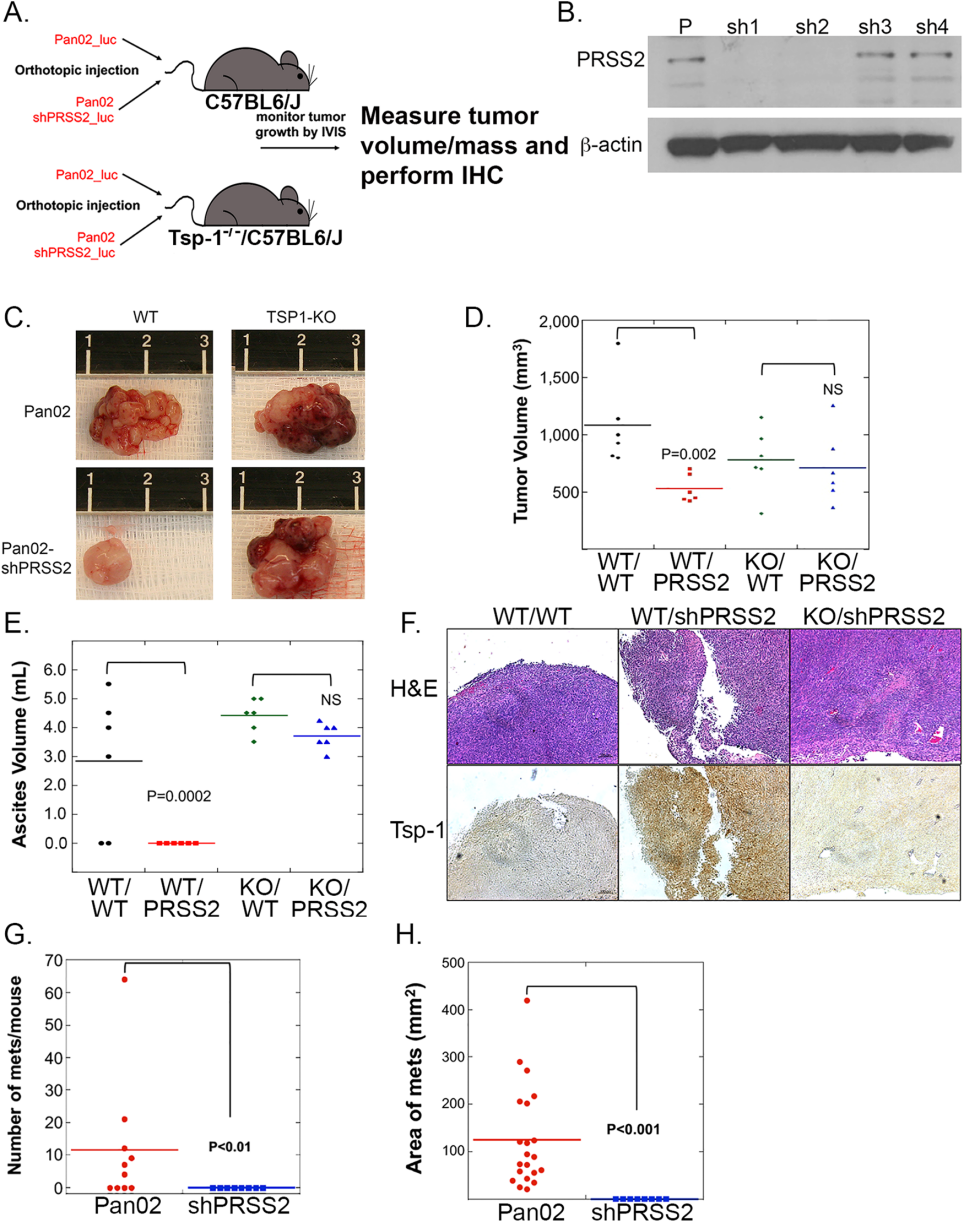
Silencing PRSS2 inhibits primary pancreatic tumor growth and metastasis. **A** Schematic of tumor implantation strategy. **B** Western blot of PRSS2 and actin in Pan02 cells transduced with lentiviral vectors specifying 4 shRNA sequences against murine PRSS2. **C** Photographs of tumors formed by Pan02 and Pan02shPRSS2 (sh1 from 4B) cells in wild-type C57Bl6/J mice and *thbs1*^−*/−*^ C57Bl6/J mice. **D** Dot plot of mass of tumors formed by Pan02 and Pan02shPRSS2 cells in wild-type C57Bl6/J mice and *thbs1*^−*/*−^ C57Bl6/J mice (*n* = 6 per group; *P* value calculated via two-sided Mann–Whitney *U* test). **E** Dot plot of ascites volume of tumors formed by Pan02 (WT *n* = 6; *thbs1*^*−/−*^ *n* = 6) and Pan02shPRSS2 (WT *n* = 6; *thbs1*^*−/−*^ *n* = 6) cells in wild-type C57Bl6/J mice and *thbs1*^*−/−*^ C57Bl6/J mice (*P* value calculated via two-sided Mann–Whitney *U* test). **F** H&E and Tsp-1 immunohistochemical staining of tumors formed by Pan02 and Pan02shPRSS2 cells in wild-type C57Bl6/J mice and *thbs1*^−*/−*^ C57Bl6/J mice (*n* = 6 mice per group) (scale bar = 200 μm). **G** Dot plot of the number of macrometastatic lesions identified by gross examination of the peritoneal cavities of mice bearing tumors formed by Pan02 and Pan02shPRSS2 cells in wild-type C57Bl6/J mice (4/6 refers to the number of mice that developed macrometastatic lesions) (*n* = 6 mice per group; *P* value calculated via two-sided Mann–Whitney *U* test). **H** Dot plot of the area of macrometastatic lesions (in mm^2^) measured using Image-J (Fiji) analysis of images of the peritoneal cavities of mice bearing tumors formed by Pan02 and Pan02shPRSS2 cells in wild-type C57Bl6/J mice (*n* = 6 mice per group: *P* value calculated via two-sided Mann–Whitney *U* test).

Based on the observation that silencing PRSS2 in SUM159 cells significantly inhibited tumor growth, we asked whether this was due to the inability to repress Tsp-1 in the TME. We postulated that in the absence of Tsp-1, silencing PRSS2 should have little to no effect on tumor growth (Fig. 5A). To test this hypothesis, we made use of Tsp-1^−/−^ mice and silenced PRSS2 in the syngeneic C57B6/J-derived pancreatic cancer cell line, Pan02 with 4 unique shRNA sequences^44^ (Fig. 5B and Supplementary Fig. S8). We found that two of the four shRNAs were effective at silencing PRSS2 expression by >95% such that no detectable protein was discernible by western blot (Fig. 5B). We examined the effect of PRSS2 in pancreatic tumor growth and progression due to the reports that PRSS2 overexpression is a contributing factor to pancreatitis, which is a precursor lesion for pancreatic cancer^45,46^. Specifically, we injected 5 × 10^5^ vector control Pan02 and Pan02shPRSS2 cells orthotopically into the head of the pancreas of wt and Tsp-1^−/−^ C57Bl6/J mice (*n* = 6 mice per cohort). We monitored tumor growth via in vivo luciferase imaging and euthanized all mice when the wt mice injected with vector control Pan02 cells became moribund due to ascites development at 28 days post injection (Fig. 5C–E). We then measured the volume and mass of all tumors and found that in wt mice, the tumors formed by Pan02shPRSS2 cells were <50% the size of vector control Pan02 tumors (528 mg vs 1082 mg; *P* = 0.002, by Mann–Whitney *U* test) (Fig. 5C, D). Conversely, in Tsp-1^−/−^ mice, the difference in the size of tumors formed by Pan02shPRSS2 cells compared to tumors formed by vector control Pan02 cells was not statistically significant (711 mg vs 779 mg; *P* = 0.588 by Mann–Whitney *U* test) (Fig. 5D). We also stained all tumors for Tsp-1 expression via immunohistochemistry and found that in wt mice, vector control Pan02 tumors had little to no detectable Tsp-1 expression, while Pan02shPRSS2 tumors had significantly higher Tsp-1 levels (Fig. 5F). As expected, all of the tumors in Tsp-1^−/−^ mice had little to no detectable Tsp-1 by IHC (Fig. 5F).

Finally, upon gross examination of mice at the time of necropsy, we observed that 6/6 mice injected with Pan02 vector control cells developed macrometastases on the wall of the peritoneal cavity and diaphragm, while 0/6 mice injected with Pan02shPRSS2 cells developed detectable metastases (Fig. 5G, H). These findings demonstrate that loss of PRSS2 significantly inhibits primary tumor growth and metastasis. However, in the absence of Tsp-1, loss of PRSS2 does not inhibit tumor growth, indicating that the major role of PRSS2 in tumor growth is the paracrine-mediated repression of Tsp-1.

### Myeloid-specific genetic deletion of LRP1 prevents repression of Tsp-1 by PRSS2

**Fig. 6:**
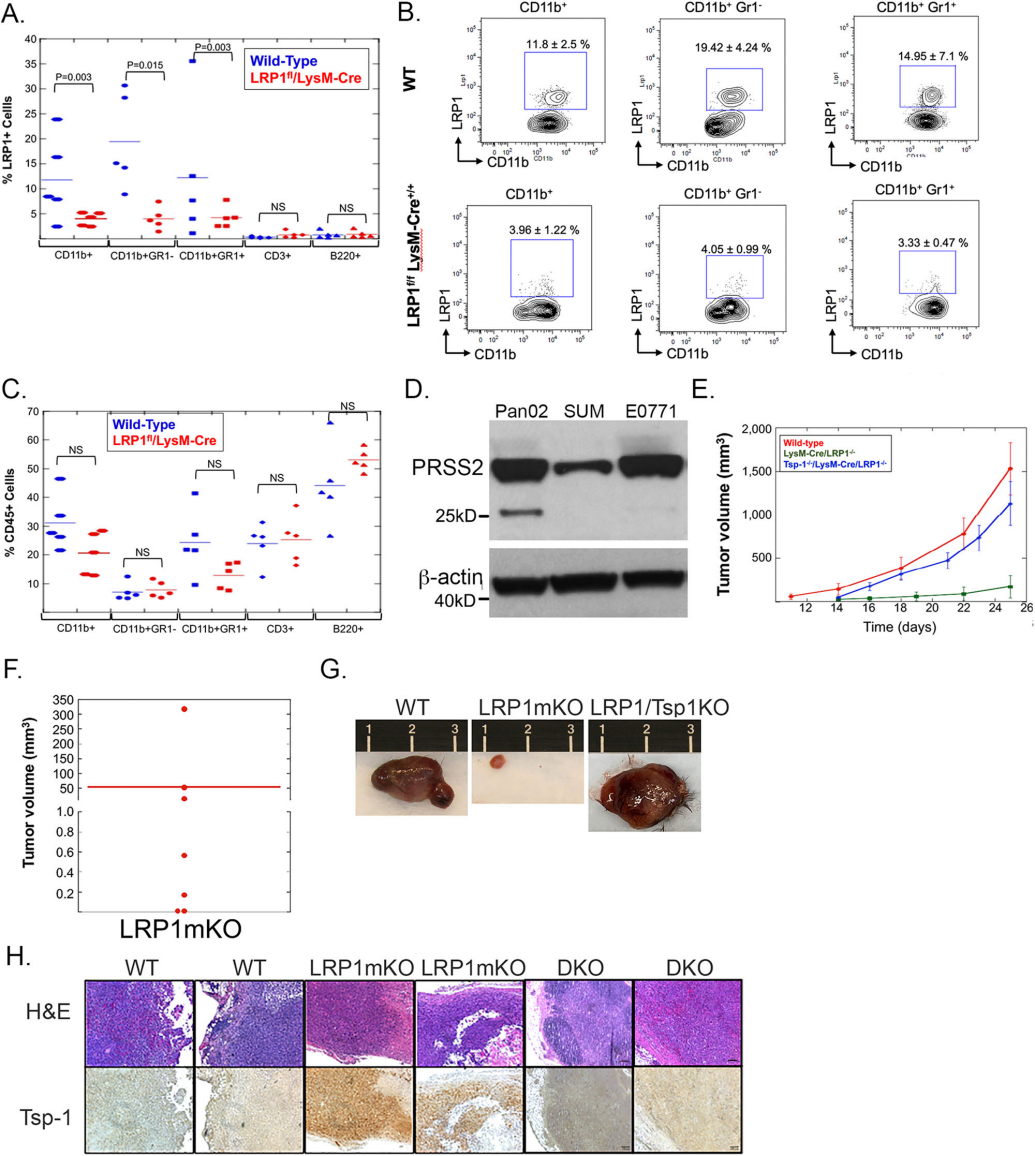
Myeloid-specific deletion of LRP1 inhibits tumor growth by preventing the repression of Tsp-1. **A** Plot of percentage of LRP1 + multiple myeloid and lymphoid cells in wild-type (*n* = 5) and LysM-Cre/LRP1^fl/fl^ (*n* = 5) mice as determined by FACS analysis. **B** Representative FACS plots of LRP1 expression in myeloid and lymphoid cells in wild-type and LysM-Cre/LRP1^fl/fl^ mice (*n* = 5 per group). **C** Plot of relative abundance of CD45 + myeloid and lymphoid cells in wild-type (*n* = 5) and LysM-Cre/LRP1^fl/fl^ (*n* = 5) mice as determined by FACS analysis. **D** Western blot of PRSS2 and Actin in Pan02 murine pancreatic cancer cells, SUM159 human breast cancer cells, and E0771 murine breast cancer cells (*n* = 3). **E** Plot of average tumor volume (as measured by calipers) of orthotopic mammary tumors formed by E0771 murine breast cancer cells in wild-type (red line), LysM-Cre/LRP1^fl/fl^ (green line), and LysM-Cre/LRP1^fl/fl^/THBS1^−/−^ (blue) mice (*n* = 7 per group) (error bars depict SEM). **F** Dot plot of volume of tumors formed by E0771 cells in LysM-Cre/LRP1^fl/fl^ mice (LRP1mKO) (*n* = 7 mice per group). **G** Photographs of tumors formed by E0771 murine breast cancer cells in wild-type, LysM-Cre/LRP1^fl/fl^, and LysM-Cre/LRP1^fl/fl^/THBS1^−/−^ mice. **H** H&E and Tsp-1 immunohistochemical staining of tumors formed by E0771 murine breast cancer cells in wild-type, LysM-Cre/LRP1^fl/fl^, and Tsp-1^−/−^/LysM-Cre-LRP1^fl/fl^ mice (*n* = 7 mice per group) (scale bars = 100 μm).

We have previously demonstrated that genetic deletion of Tsp-1, in the entire mouse or specifically in bone marrow-derived cells, abrogates the ability of prosaposin (PSAP) to inhibit tumor growth^4,5^. Accordingly, we specifically deleted LRP1 in myeloid-derived cells by crossing LysM-Cre mice with LRP1^flox/flox^ mice (Supplementary Fig. S9A). FACS analysis revealed that LRP1 is predominantly expressed by CD11b + myeloid cells, as opposed to T (CD3 + ) and B (B220 + ) cells (Fig. 6A, B). Importantly, myeloid-specific knockout of LRP1 did not affect production of myeloid cells in these mice (Fig. 6C and Supplementary Fig. S10).

We then sought to determine whether the disruption of PRSS2-LRP1 signaling in myeloid cells could inhibit primary tumor growth. We injected wild-type C57BL6/J mice and LysM-Cre-LRP1^−/−^ mice with the murine triple-negative breast cancer cell line E0771, which express similar levels of PRSS2 to Pan02 cells and even greater levels than SUM159 cells (Fig. 6D). Strikingly, we found that tumors formed by E0771 tumors in the myeloid-specific LRP1 knockout mice were 28.3-fold smaller than tumors formed in wild-type mice (54.9 mm^3^ vs 1,531 mm^3^; *P* = 0.002 by Mann–Whitney *U* test) (Fig. 6E, G). Moreover, four out of seven tumors that formed in LysM-Cre-LRP1^−/−^ mice were less than 2 mm in diameter (Fig. 6F). Histologic analysis of the tumors that formed in the LysM-Cre-LRP1^−/−^ mice revealed abundant Tsp-1 in the TME, while tumors in wt C57BL6/J mice had virtually undetectable levels of Tsp-1 (Fig. 6H). To confirm that the loss of LRP1 inhibited tumor growth due to the inability of tumors to repress Tsp-1, we crossed the LysM-Cre-LRP1^−/−^ with Tsp1^−/−^ mice to generate double-knockout (DKO) mice that lacked Tsp-1 globally and LRP1 in myeloid cells (Supplementary Fig. S9B). We injected 1 × 10^6^ E0771 cells into the mammary gland of the DKO mice and monitored tumor growth by direct measurement. We found that the average size of E0771 tumors that formed in the DKO mice ranged between 75 and 93% of the size of the tumors that formed in wild-type mice. Moreover, tumors that formed in the DKO mice had undetectable levels of Tsp-1 by IHC, as expected (Fig. 6E–H). These findings indicate that LRP1 on myeloid cells is required for the PRSS2-mediated repression of Tsp-1 in the TME and that the inability to repress Tsp-1 in the TME significantly impairs tumor growth.

### Repression of Tsp-1 creates an immunosuppressive tumor microenvironment

Given the profound inhibitory effects of silencing PRSS2 on tumor growth and progression, we examined whether modulating Tsp-1 expression in the TME had any effect on the tumor immune landscape. It has been demonstrated that regulatory T cells (Tregs) express high levels of CD36, which makes them metabolically dependent on free fatty acid uptake^47^. Tsp-1 binds to CD36 and blocks fatty acid uptake, in addition to inducing apoptosis^48,49^. Thus, we postulated that tumors in which PRSS2 was silenced, and consequently express high levels of Tsp-1, would have fewer Tregs and a higher ratio of CD8 + T cells to Tregs. Accordingly, we analyzed Pan02 and Pan02shPRSS2 tumors that formed in WT and Tsp-1^−/−^ mice for T-cell markers.

**Fig. 7:**
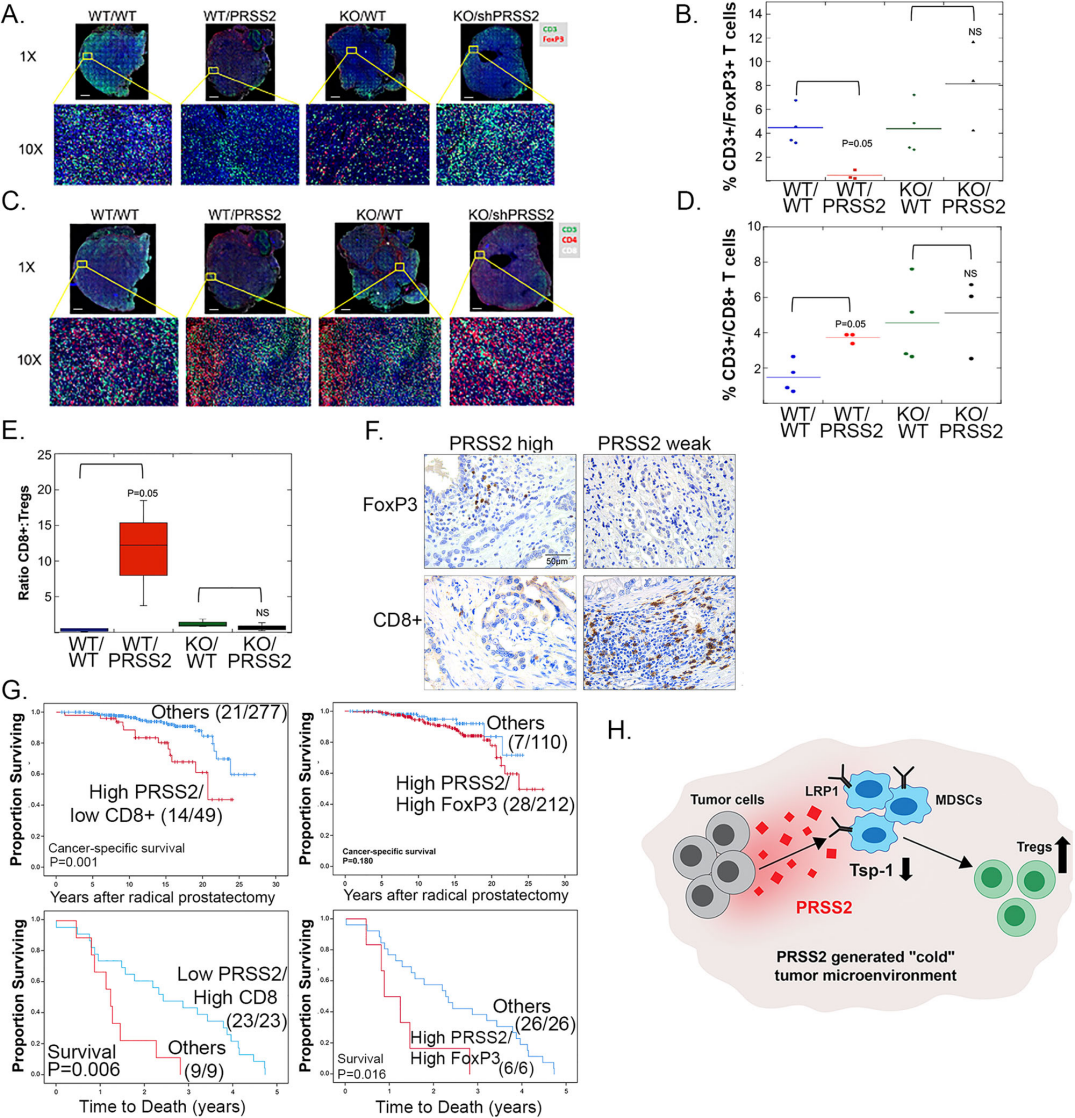
Repression of Tsp-1 by PRSS2 creates and immunosuppressive TME. **A** Immunofluorescence staining of CD3 (green) and FoxP3 (red) in tumors formed by Pan02 (*n* = 4) and Pan02shPRSS2 (*n* = 3) cells in WT C57Bl6/J mice and *Tsp1*^*−/−*^ C57Bl6/J mice (scale bar = 1 mm). **B** Plot of the percentage of CD3 + /FoxP3 + T cells out of total CD3 + T cells in tumors formed by Pan02 (*n* = 4) and Pan02shPRSS2 (*n* = 3) cells in WT C57Bl6/J mice and *thbs1*^−*/−*^ C57Bl6/J mice (*P* value calculated via two-sided Mann–Whitney *U* test). **C** Immunofluorescence staining of CD3 (green), CD4 (red), and CD8 (white) in tumors formed by Pan02 (*n* = 4) and Pan02shPRSS2 (*n* = 3) cells in WT C57Bl6/J mice and *Tsp1*^*−/−*^ C57Bl6/J mice (scale bar = 1 mm). **D** Plot of percentage of CD3 + /CD8 + T cells out of total CD3 + T cells in tumors formed by Pan02 (*n* = 4) and Pan02shPRSS2 (*n* = 3) cells in WT C57Bl6/J mice and *thbs1*^*−/−*^ C57Bl6/J mice (*P* value calculated via two-sided Mann–Whitney *U* test). **E** Plot of ratio of CD3 + /CD8 + T cells to CD3 + /FoxP3 + T cells in tumors formed by (*n* = 4) and Pan02shPRSS2 (*n* = 3) cells in WT C57Bl6/J mice and *thbs1*^*−/*−^ C57Bl6/J mice (WT/WT minimum = 0.099; maximum = 0.58; median = 0.37; percentile = 25–75; *P* value calculated via two-sided Mann–Whitney *U* test). **F** Representative pictures of immunohistochemistry of CD8 + and FoxP3 + T cells in prostate tumors (*n* = 458 patients) with high or weak levels of PRSS2 (scale bar = 50 μm). **G** Kaplan–Meier curves of: upper left: correlation of high PRSS2 and low CD8 levels with survival after radical prostatectomy. Upper right: correlation of high PRSS2 and high FoxP3 levels with survival after radical prostatectomy; and lower left: correlation of low PRSS2 and high CD8 levels with survival after developing castration resistance; and lower right: correlation of high PRSS2 and high FoxP3 levels with survival after developing castration resistance (*P* values calculated via two-sided Mann–Whitney *U* test). **H** Schematic diagram of PRSS2 regulation of tumor immune microenvironment.

We performed whole slide immunohistochemical analysis of total T cells (CD3 +), CD4 + T cells, CD8 + T cells, and FoxP3+ Tregs. In WT mice, we observed a ninefold decrease of Tregs in Pan02shPRSS2 tumors compared to control Pan02 tumors (4.5% to 0.48% of all T cells; *P* = 0.05, by Mann–Whitney *U* test) (Fig. 7A, B). Strikingly, in Tsp-1^−/−^ mice the percentage of Tregs did not differ significantly between Pan02 and Pan02shPRSS2 tumors (4.4% vs 6.95%, *P* = 0.63, by Mann–Whitney *U* test) (Fig. 7A, B). Moreover, in WT mice, the percentage of CD3 +/CD8 + T cells in tumors formed by Pan02shPRSS2 cells was >twofold higher than in vector control Pan02 tumors (3.36% vs 1.59%; *P* = 0.057 by Mann–Whitney *U* test) (Fig. 7C, D). Conversely, in Tsp-1^−/−^ mice there was no significant difference in CD3/CD8 + T cells between Pan02shPRSS2 and vector control Pan02 tumors (3.97% vs 5.1%, *P* = 0.7 by Mann–Whitney *U* test) (Fig. 7C, D). Thus, the ratio of CD8 + :FoxP3 + T cells was 20-fold higher in tumors formed by Pan02shPRSS2 cells than in tumors formed by vector control Pan02 cells (3.59:0.48 = 7 vs 1.59:4.5 = 0.35) (Fig. 7E). These findings indicate that the repression of Tsp-1 results in the generation of an immunosuppressive tumor microenvironment with high levels of regulatory T cells and a low ratio of CD8 + :Treg cells. Moreover, our results indicate that blocking the repression of Tsp-1 potently increases CD8 + T cells and concomitantly reduces Treg infiltration which could result in a more active tumor immune microenvironment.

To further examine the effects of PRSS2 on the immune microenvironment, we analyzed the infiltration of CD8 + and FoxP3 + T lymphocytes and their correlation with PRSS2 expression in patient tumor tissue samples. We found that in clinical primary tumor tissues, high levels of PRSS2 expression were associate with increased infiltration of FoxP3+ Tregs in radical prostatectomy specimens (Fig. 7F and Table 2). Consistent with these findings, levels of CD8 + T lymphocytes, were inversely correlated with PRSS2 expression (Fig. 7F and Table 2). Strikingly, these associations were also significantly tied to outcome. Specifically, prostate cancer patients with low CD8 +/strong PRSS2 expression or high FoxP3 +/strong PRSS2 expression had significantly poorer outcome (Fig. 7G, H and Supplementary Fig. S11). Taken together these findings indicate that PRSS2 creates an immunosuppressive TME, characterized by high levels of FoxP3 Tregs and low levels of CD8 + T cells.

## Discussion

The ability of tumor cells to modify their microenvironment to create a site that is permissive for growth is a key factor in distinguishing aggressive, metastatic tumors from localized lesions. It has been previously reported that the tumor cell-autonomous repression of Tsp-1 is required for tumors to escape dormancy^42,43^. Here, we demonstrate that repression of Tsp-1 in the tumor microenvironment is also required for tumors to escape dormancy.

In this report, we describe the identification of a novel stimulator of tumor growth and metastasis, the serine protease PRSS2. Of note, we demonstrate that the tumor growth-promoting activity of this protein lies not in its enzymatic activity but in its ability to act as a ligand for the cell surface receptor LRP1. In doing so we also discovered a previously unknown biologic activity for the LRP1/Rac pathway, the repression of the anti-angiogenic, anti-tumorigenic, and anti-inflammatory protein Tsp-1. While we demonstrate a direct interaction between domain 1 of LRP1 and PRSS2 and that LRP1 is required for the repression of Tsp-1 by LRP1, we cannot at this time rule out the involvement of a co-receptor for LRP1 in this process. Of additional interest is the relationship between Tsp-1 and LRP1 as LRP1 has been shown to be an endocytic receptor for Tsp-1^50,51^ and thus our findings could indicate a potential feedback mechanism between Tsp-1 production and LRP1 signaling.

Silencing PRSS2 in tumor cells significantly attenuates tumor growth and metastasis. In addition, silencing PRSS2 also resulted in a TME that is less immunosuppressive, as evidenced by increased infiltration of CD8 + T cells and decreased infiltration of Tregs resulting in an increased ratio of CD8:Treg. Critically, these alterations in immune cell composition of the TME were Tsp-1-dependent as they were not observed in Tsp-1^−/−^ mice. Moreover, specific genetic deletion of the receptor for PRSS2, LRP1, in myeloid cells recapitulates the tumor-inhibitory effects of silencing PRSS2. Finally, the observations made in pre-clinical models were recapitulated in observations made in clinical patient tumor samples.

Critically, the experimental findings that PRSS2 is required for efficient tumor growth were validated by the observation that PRSS2 expression correlates with aggressive clinical features such as angiogenesis, tumor cell proliferation, disease progression, and survival in prostate cancer patients. When the expression of CD8 or FoxP3 on T cells was considered there was a strong correlation between high PRSS2 and high FoxP3 with decreased overall survival. Conversely, we found a strong correlation between low PRSS2 and high CD8 expression with increased overall survival. These findings are consistent with previous reports that LRP1 tethers Tsp-1 to the surface of T cells to enhance adhesion and increase TCR-induced T-cell activation^52^. Thus, induction, or inhibition of repression, of Tsp-1 in the TME enhances CD8 + T-cell infiltration and decreases FoxP3+ Treg infiltration. Conversely, repression of Tsp-1 expression by PRSS2 inhibits CD8 + T-cell infiltration and augments FoxP3+ Treg infiltration.

We previously demonstrated that tumors that express high levels of prosaposin (PSAP) remain localized and metastasize with very low frequency due to the induction of Tsp-1 expression in the TME^4,5,16^. The progression, or lack thereof, of tumors could thus potentially be explained as a competition between PRSS2 and PSAP. If tumors make more PSAP than PRSS2 they will grow more slowly, and if tumors make more PRSS2 than PSAP they will progress more rapidly. This hypothesis was born out by the observations that PRSS2 expression negatively correlates with PSAP expression in prostate cancer.

Based on the findings presented in this report, we predict that therapeutic strategies, which augment PSAP activity and inhibit PRSS2 binding to LRP1 could hold tremendous therapeutic potential for cancer patients. Significantly, this strategy should have relatively few adverse effects, as the therapeutic agents would not have direct cytotoxic activity. Moreover, since these therapeutic agents would target biological mechanisms in genetically normal cells that comprise the tumor microenvironment they would be less likely to induce resistance and potentially be indication agnostic.

## Methods

### Cell culture and siRNA transfection

WI38 cells were cultured in Minimal Essential Media (MEM), containing 10% fetal bovine serum (FBS) and 1% penicillin/streptomycin at 37 °C. HEK293T cells were seeded the day before the experimentation and were transiently transfected with pCMV-SPORT6-PRSS2 (Harvard Plasmid Repository) at a density of 80% confluence using Lipofectamine 3000 according to the manufacturer’s protocol (Invitrogen). For siRNA transfection, WI38 cells were transiently transfected with 25 nM siLRP1 (Sigma) using Lipofectamine 3000 according to the manufacturer’s protocol (Thermo Fisher). Gene silencing was confirmed by immunoblotting 72 h post transfection. WI38 cells were a gift of the Weinberg Lab (MIT). The murine pancreatic adenocarcinoma cell line (PAN02, also known as Panc02) was purchased from the Developmental Therapeutics Program, Division of Cancer Treatment and Diagnosis, National Cancer Institute (Frederick, MD). E0771 cells purchased from ATCC were cultured in DMEM plus 10% FBS. SUM159 cells were cultured in Ham’s F12 media plus 10% FBS and were a gift of Stephen Ethier (University of South Carolina Medical College). Primary human peripheral blood mononuclear cells were purchased from Lonza and cultured in X-VIVO^TM^ 20 Serum-free Hematopoietic Cell Medium (Lonza).

### Treatment of cells with chemical inhibitors and recombinant protein

WI38 cells were seeded 24 h before the treatment and synchronized for 2 h in a serum-free medium. Synchronized cells were treated with 25 µm Rac1 inhibitor NSC23766 (Millipore), 10 µm JNK inhibitor III (Millipore), 25μM Nutlin-3 (Sigma-Aldrich), or PRSS2 conditioned medium for 45 min or 16 h and subjected to immunoblot analysis.

### Western blot analysis

Western blot was performed as described previously^5^. The following primary antibodies were used: Tsp-1 (rabbit pAb, Abcam), LRP1 (rabbit pAb, Cell Signaling), pJNK (Thr183/Tyr185) (rabbit mAb, Cell Signaling), JNK (rabbit mAb, Cell Signaling), Phospho-c-Jun (Ser73) (rabbit mAb, Cell Signaling), c-Jun (rabbit mAb, Cell Signaling), β-actin (mouse mAb AC-15, Abcam), PRSS2 (ab223064, Abcam).

### Generation of PRSS2 mutants

Single mutation of PRSS2 in pCMV-Sport6 vector (Harvard Plasmid Repository) (G191R, S200A, S200C and S200T) were introduced by site-directed mutagenesis using QuikChange Lightning Site-Directed Mutagenesis Kit according to manufacturer’s protocol (Agilent Technologies). To express PRSS2 WT and Mutants protein, transient transfections were carried out in HEK293 cells cultured in a six-well culture plate using Lipofectamine 3000 and 2 µg of PRSS2 wild-type or mutations were used. The transfection medium was replaced with serum-free DMEM medium 48 h after transfection, and conditioned medium containing secreted PRSS2 WT and mutation protein was collected after an additional 24 h.

### PRSS2 enzymatic activity assay

Recombinant PRSS2 and mutation protein was overexpressed in HEK293 cells as described above. Cells were cultured in DMEM phenol red-free medium to reduce background reading for fluorescence assay. Conditioned medium (100 µl) was supplemented with 0.1 M Tris-HCl (pH 8.0), 1 mM CaCl_2_ (final concentrations). PRSS2 activity were determined with 21.5 µM Mca-RPKPVE-Nval-WRK(Dnp)-NH_2_ Fluorogenic MMP Substrate (R&D Systems) (final concentration) and incubated at 25 °C. The activity was measured every 10 min for total 70 min and expressed as a percentage of potential total enzymatic activity. Triplicate experiments were performed for each construct.

### shRNA-mediated silencing of PRSS2 and Myc

shRNA lentiviral constructs (pLKO) for human Myc and human and mouse PRSS2 were purchased from Sigma-Aldrich. The shRNA sequences against human PRSS2 were: (1) CCCTGGAAAGATTACCAACAA, (2) CCCAAATACAACAGCCGGACT, (3) GCTACATCTGTGAGGAGAATT. The shRNA sequences against murine PRSS2 were: (1) AGAACACAATTGCTGACAACT, (2) CCCTATCAGGTGTCCCTAAAT, (3) CCTCAGCAATGGTGTGAACAA, (4) CAATTATAATTCATGGACCCT. The shRNA sequences against human Myc were: (1) CAGGAACTATGACCTCGACTA, (2) CCTGAGACAGATCAGCAACAA, (3) GTGCAAGTTGGACATTACAAT.

Lentiviral particles were generated by transfecting HEK293T cells and transferring the conditioned media to SUM159 or Pan02 cells, followed by selection with 1 μg/mL puromycin. Knockdown was confirmed by western blot analysis and RT-PCR.

### Reverse-transcriptase real-time PCR

The knockdown level of PRSS2 by shRNA in Pan02 cells was measured by real-time quantitative PCR. The following primers were used: mPRSS2_F 5′-GCTCACTGCTACAAATACCGCATCCA-3′, mPRSS2_R 5′-AGGTACAGAGGCCACTCTGGCATTG-3′. RNA was extracted from each sample cell line using RNeasy Mini Kit (Qiagen). And 1 µg of RNA from each sample was converted to double-stranded cDNA using cDNA synthesis Kit (Qiagen). RT-PCR was performed using SYBR Green Master Mix (Applied Biosystems) on StepOne™ Real-Time PCR System (Thermo Fisher).

### Co-immunoprecipitation

WI38 cells from 15-cm dishes were collected and lysed in lysis buffer (25 mM Tris (pH 7.2), 150 mM NaCl, 5 mM MgCl2, 0.5% NP-40, 1 mM DTT, 5% glycerol) containing fresh protease inhibitor and phosphatase inhibitor cocktail (Thermo Fisher), followed by centrifugation to remove cellular debris. Lysates were pre-cleared by incubating with 20 µl protein A/G agarose suspension for 1 h at 4 °C. The pre-cleared lysates were incubated with 10 µg PRSS2 antibody (Abcam) overnight at 4 °C, followed by incubation with 60 µl protein A/G agarose suspension for 3 h at 4 °C. Agarose beads were then collected by centrifugation, washed four times with 50 mM Tris, 150 mM NaCl, and 0.1% Tween-20, and resuspended in 100 µl RIPA buffer containing 6× SDS sample buffer. Samples were boiled for 5 min and the supernatant were collected after centrifugation for mass spectrometry analysis.

### Rac1 and RhoA GTPase activation assay

Rac1 and RhoA GTPase activation was measured by G-LISA Rac1 activation assay Kit and G-LISA RhoA activation Kit (Cytoskeleton), according to the manufacturer’s protocol. Briefly, WI38 cells were serum-starved for 2 h, and the 293T conditioned medium with pCMV (control vector) and pCMV_PRSS2 vector overexpression was added to the serum-starved cells for 45 min. The cells are washed with cold PBS and lysed in ice-cold lysis buffer containing protease inhibitors. Protein concentrations were measured, and the same amount of protein was used under each condition. The cell lysates were incubated in Rac1 and RhoA assay wells along with blank control and positive control for 30 min at 4 °C with agitation at 400 rpm. The wells were washed with Wash Buffer followed by incubation with Antigen Presenting Buffer at room temperature. The primary and secondary antibodies were incubated at room temperature for 45 min. The HRP detection reagent were incubated in each well for exact 3 min and the luminescence signal were detected using a microplate luminescence reader.

### Pre-clinical human and murine breast cancer models

All animal work was conducted in accordance with a protocol approved by the Institutional Animal Care and Use Committee (IACUC) of Boston Children’s Hospital. Female SCID mice (*n* = 8/ group) (6–8 weeks old) were purchased from Mass General Hospital. For orthotopic breast cancer cell injection, SUM159 vector-luc and SUM159shPRSS2-luc cells were injected into the mammary fat pad of the female SCID mice (1 × 10^6^ cells/20 μl). The tumor burden was monitored weekly by both bioluminescence imaging using Xenogen IVIS system and tumor size measurement by caliper.

Myeloid-specific LRP1 knockout mice were generated by crossing LysM-Cre mice with LRP1^flox/flox^ mice. Murine triple-negative breast cancer cell line E0771 (1 × 10^6^ cells/ 20 μl) were orthotopically injected into the mammary fat pad of the female LysM-Cre-LRP1^−/−^ mice (*n* = 8/group) (6 weeks old) and wild-type C57BL6/J mice (*n* = 8/group) (6 weeks old). The tumor size was measured twice a week by caliper.

LRP1 and Tsp-1 double-knockout (DKO) mice were generated by crossing LysM-Cre-LRP1^−/−^ with Tsp-1^−/−^ mice. The double knockout was confirmed by genotyping using the following primers:

Lrp1^flox^_F: CATACCCTCTTCAAACCCCTTCCTG,

Lrp1^flox^_R: GCAAGCTCTCCTGCTCAGACCTGGA,

Tsp1_F: GAGTTTGCTTGTGGTGAACGCTCAG,

Tsp1wt_R: AGGGCTATGTGGAATTAATATCGG,

Tsp1ko_R: TGCTGTCCATCTGCACGAGACTAG.

E0771 cells (1 × 10^6^ cells/ 20 μl) were orthotopically injected into the mammary fat pad of the female DKO mice (*n* = 10/group) (6 weeks old). The tumor size was measured twice a week by caliper.

### Pre-clinical murine pancreatic tumor model

Thrombospondin-1 deficient mice (Tsp-1^−/−^, *n* = 8/group) were purchased from the Jackson Laboratory (#006141) and maintained by mating Tsp-1^−/−^ males with Tsp-1^−/−^ females. C57BL6/J mice (*n* = 8/group) (8 weeks old) were purchased from the Jackson Laboratory (#000664) and used as Tsp-1^+/+^ controls. For orthotopic pancreatic cancer cell injection, pancreatic cell lines wtPan02_luciferase and Pan02shPRSS2_luciferase (5 × 10^5^ cells/10 μl), washed and harvested in HBSS and mixed 1:1 with Matrigel, were injected into the tail of the pancreas (20 μl total volume). The tumor burden was determined weekly by bioluminescence imaging using Xenogen IVIS system initiated 7 days post injection.

### Pre-clinical prostate cancer xenograft model

Male SCID mice 6–8 weeks old were injected with 2 × 10^6^ viable PC3 or PC3M-LN4 human prostate cancer cells in the prostate gland in a volume of 10 μL. The cells were washed and harvested in PBS prior to injection into the prostate glands of anesthetized mice (2% avertin, 0.5 ml per mouse). Endpoint assays were conducted at 5 weeks after injection unless significant morbidity required that the mouse be euthanized earlier. Tumors were harvested following euthanasia and stained for Tsp-1 as described below.

### Thrombospondin-1 immunohistochemistry

Tumor samples were fixed in 10% paraformaldehyde and subsequently paraffin-embedded for sectioning. The paraffin-sectioned slides were deparaffinized with xylene and rehydrated in decreasing concentration of ethanol to water. For Thrombosponsdin-1 staining, the antigen retrieval was performed with proteinase K (Roche Diagnostics) at a final concentration of 20 μg/ml in 0.2 M Tris pH 7.2 at 37 °C for 25 min. The slides were then blocked with 2.5% goat serum (Vector Laboratories) for 30 min at room temperature. Slides were incubated overnight at 4 °C with primary antibody rabbit anti-Thrombospondin-1 (ab226383, Abcam) The slides were then washed in PBS with 0.05% Tween (three times for 5 min), followed by incubation with HRP-conjugated secondary antibody (Vector Laboratories) for 30 min at room temperature. Slides were then incubated with DAB substrate (Vector Laboratories) followed by counterstain with Hematoxylin (Vector lab, H3401).

### T-cell Immunohistochemistry in pre-clinical models

Immunohistochemistry (IHC) was performed as previously described^53^. Briefly, slides were warmed in a 60 °C oven for 10 min followed by deparaffinization and rehydration. Before antigen retrieval, slides were fixed in 10% neutral buffered formalin for 30 min. Antigen retrieval was performed in antigen retrieval buffer (10 mM Tris-HCl, 1 mM EDTA with 10% glycerol [pH 9]) at 110 °C for 17 min (∼4–5 ψ). Slides were then allowed to be cooled down to room temperature and were washed once with PBS. Tissue sections were blocked with 2.5% goat serum (Vector Laboratories, S-1012) for 30 min followed by incubation with primary antibody overnight: CD4 (1:1000; Abcam, ab183685), Foxp3 (1:200; R&D Systems, MAB8214). After washing, the slides were incubated with HRP-conjugated secondary Antibody (ImmPRESS; Vector Laboratories, MP-7401) for 30 min on a shaker. For developing the fluorescence signal, TSA detection system (PerkinElmer) was used. We used OPAL 520, OPAL 570 and OPAL 690 fluorophores for staining the different markers. Multiplex staining was performed by stripping the previous antibody in 10 mM citrate buffer (pH 6.2) plus 10% glycerol at 110 °C for 2 min before probing with the next primary antibodies in the next two consecutive rounds: CD3 (1:2000; Thermo Fisher Scientific, PA1-29547), and CD8 (1:4000; Cell Signaling, 98941). Slides were counterstained with DAPI, and then cover slipped using ProLong Gold mount (no. P36931; Life Technologies). Slides were scanned at 20X using the Zeiss AxioScan.Z1 (Whole Brain Microscopy Facility, UT Southwestern). The following channels were used to acquire images: DAPI, AF488 (for OPAL 520), AF555 (for OPAL 570), and AF660 (for OPAL 690).

### Integrative data analysis

To analyze immune cell infiltration, stained images were processed to extract cell coordinates and analyze their numbers. The following sets of stained were processed: DAPI + CD3 + CD4 + CD8 and DAPI + CD3 + Foxp3. QuPath (ver. 0.2) software was used to load whole scan images and detect cells in the whole tissue^54^. Cells were detected by using the standard implemented routine over the DAPI channel that stains cell nuclei. The default detection parameters were used, except the background radius was set to 20 µm and cell expansion −2 µm. The size of the background was set to be larger than cell nuclei. The cell expansion parameter, which extrapolates cell boundaries based on nucleus shape, was limited to 2 µm because tumor tissue possesses tight packing of nuclei (cells) making cell co-staining information (e.g., CD8, F480) more specific and reducing artefacts. The detection results, including cartesian coordinates of cell nuclei and mean staining intensity in each cell area, we saved into text files for further processing.

The cell phenotypes were then assigned based on staining intensity in the individual channel to identify double-positive cells as CD3CD4, CD3Foxp3, etc. A cell was counted positive if it was above the threshold, which was estimated from an intensity distribution individually for each image per channel. Intensity histogram was calculated based on all cells revealing a gaussian-like distribution with longer right-shoulder, where gaussian distribution was attributed to the background signal and larger intensities for positive cells. Gaussian fit was performed over the peak estimating the average value, *m*, and the standard deviation, s.d. Then cells were determined to be positive if their intensity in their corresponding channel (marker) was larger than m + 3*s.d., i.e., the positive signal was three standard deviations above the background signal (Supplementary Fig. S12). Such a procedure enabled to reduce human bias. Only CD4 threshold was adjusted manually, because of stronger staining artefacts in the background. The double-positive cell, e.g., CD3CD4 (Supplementary Fig. S13), had corresponding cell intensities larger than the corresponding threshold values.

### Breast cancer patient series

Two independent breast cancer series were immunohistochemically stained for PRSS2 protein. Series 1 is a population-based cohort of 544 primary breast carcinomas from the period 1996 to 2003, and Series 2 is a case–control series of 202 primary breast carcinomas (53 *BRCA1*, 45 *BRCA2* and 104 *BRCA* non-mutated) from the period 1986 to 2005, as previously described^55,56^. Twenty-six cases from Series 1 and 30 cases from Series 2 were excluded due to technical inadequate material, leaving 518 and 172 cases for evaluation of PRSS2 staining. Outcome data was only available for Series 1 and included survival time, survival status and cause of death. Last date of follow-up was June 30, 2017 (median follow-up time of survivors, 216 months; range 166–256). During follow-up, 87 patients (17%) died of breast cancer, and 199 (23%) died of other causes.

The study was approved by the Western Regional Committee for Medical and Health Research Ethics, REC West (REK 2014/1984) (Series 1) and the Institutional Review Board at McGill University Hospital, A03-M33-02A (Series 2). All studies were performed in accordance with guidelines and regulations by the University of Bergen and REK, and in accordance with the Declaration of Helsinki Principles.

### Clinico-pathologic variables

The following variables were available: age at diagnosis, tumor diameter, histologic type, histologic grade, lymph node status, hormonal (ER, PR) and HER2 receptor status, proliferation markers (Ki67, mitotic count), CK5/6 (basal marker), p53 protein expression, proliferating microvessel density (pMVD) (nestin and Ki67 co-expression, Series 1; Factor VIII and Ki67 co-expression, Series 2)^57^. The glomeruloid microvascular phenotype (GMP), a marker of increased tumor-associated angiogenesis, was available for a subset of Series 1^58^.

### PRSS2 immunostaining

Manual staining for PRSS2 in the breast cancer series was primarily performed on tissue microarray (TMA) sections, and regular sections were used in cases with poor quality or insufficient tumor material for evaluation in the TMA cores (72 cases in Series 1). The sections (5 µm) with formalin-fixed and paraffin-embedded tissue were deparaffinized with xylene, rehydrated in decreasing concentrations of alcohol and rinsed in distilled water. The slides were boiled in buffered solution at pH 6 (DAKO S1699) using a microwave oven for 20 min at 350 W. After 15 min of cooling and the addition of distilled water to reduce the fluid to room temperature, the slides were moved to a humidifying chamber (Magnetic Immuno Staining Tray, Cell Path, UK). To reduce the endogenous peroxidase, a peroxidase-blocking agent (DAKO S2023) was added for 8 min. Between the different steps, rinsing with buffered saline solution (DAKO S3006) was performed. The sections were incubated for 60 min at room temperature with the rabbit antibody PRSS2 (Center) (Sigma-Aldrich, SAB 1307060) diluted 1:50 in antibody diluent with background reducing components (DAKO S 3022). A secondary antibody (HRP EnVision rabbit (DAKO K4003) was added for 30 min at room temperature. For visualization, DAB (DAKO K3468) was used as chromogen and the slides were counterstained with hematoxylin (DAKO S3301). Multiorgan TMA sections were included as positive and negative controls. The negative controls were obtained by adding antibody diluent without the primary antibody.

### Evaluation of PRSS2 staining in tumor cells and stroma

PRSS2 staining in tumor cells was recorded using a semi-quantitative and subjective grading system, considering the intensity of staining (none = 0, weak = 1, moderate = 2, and strong = 3) and the proportion of tumor cells showing a positive reaction (<10% = 1, 10–50% = 2, >50% = 3). A staining index (values 0–9) was calculated as a product of staining intensity (0–3) and proportion of immunopositive cells (1–3)^59^. The evaluation of PRSS2 staining in the tumor stroma (tumor microenvironment staining, TME) was a combined subjective recording of the intensity of staining in cells present in the stroma compartment, mainly immune cells and fibroblasts (none = 0, weak = 1, moderate = 2, and strong = 3). If the staining intensity in stromal cells was heterogenous, the scoring was based on the predominant pattern.

As there is yet no validated cutoff value for PRSS2 expression, the distribution and frequency histograms for SI and intensity were evaluated. PRSS2 expression in tumor cells was considered low for SI 0–6 (88%) and high for SI 9 (12%) in Series 1 (upper quartile), and low for SI 0–4 (66%) and high for SI 6–9 (34%) in Series 2 (median). Stromal PRSS2 expression was considered low for staining intensity 0–2 (83% and 76%), and high for staining intensity 3 (17% and 24%) in Series 1–2.

### Statistical analysis

Data were analyzed using the SPSS Statistics for Windows, Version 25.0. (IBM Corp., Armonk, NY, USA). Statistical significance was assessed at the two-sided 5% level, whereas borderline statistical significance was defined as *P* values between 5% and 10%. Associations between categorical variables were evaluated using the Pearson’s *χ*² test or Fisher’s exact test, as appropriate, and odds ratios (OR) were computed. Univariate survival analyses were carried out using the Kaplan–Meier method with significance determined by the log-rank test. The endpoint in survival analyses was breast cancer-specific survival (BCSS) (Series 1). The entry date was the date of diagnosis. Patients who died from other causes were censored at the date of death.

### Prostate cancer

Five different malignant and benign series of prostatic tissues were used. Series 1 include carcinoma tissue from 338 patients treated by radical prostatectomy for clinically localized prostate cancer (Haukeland University Hospital, Bergen, Norway during 1986-2007). Series 2 cover 41 cases of benign prostatic hyperplasia. Series 3 include 33 non-skeletal metastases (27 lymph nodes and 6 distant soft tissue metastases [orbital, testis, skeletal muscle, rectum, subcutaneous tissue, and bronchial mucosa]). Series 4 are 13 skeletal metastases and Series 5 include tissues from 33 patients with castration-resistant prostate carcinoma receiving palliative treatment with transurethral resection of the prostate during 1990–2005.

### Clinico-pathologic variables

Clinico-pathologic information was retrieved from the clinical patient files and pathology reports for the patients in Series 1. The information included age at diagnosis, preoperative and postoperative s-PSA, clinical TNM stage, Gleason grading, largest tumor dimension, involvement of surgical margins, extra-prostatic extension, seminal vesicle invasion, and pelvic lymph node status at prostatectomy. In addition, biomarkers from previous studies, such as Ki67^60^, VEGF-A^61^, and Saposin C^4^ were included.

Follow-up information involved time from surgery until biochemical recurrence, clinical recurrence, loco-regional recurrence, skeletal metastasis and death, including prostate cancer-specific death for the patients in Series 1 and time from castration resistance to death for the patients in Series 5.

Tissue microarrays consisted of three tissue cores from each case (diameter 0.6–1.0 mm), selected from areas with the highest tumor grade. Regular slides were used for the skeletal metastases.

### PRSS2 immunostaining

Immunohistochemical staining for PRSS2 in the prostate cancer series was performed manually. Tissue microarray (TMA) sections and regular sections (5 µm) from formalin-fixed and paraffin-embedded tissue were deparaffinized with xylene, rehydrated in decreasing concentrations of alcohol and rinsed in distilled water. The slides were boiled in buffered solution at pH 6 (DAKO S1699) using a microwave oven for 20 min at 350 W. After 15 min of cooling and the addition of distilled water to reduce the fluid to room temperature, the slides were moved to a humidifying chamber (Magnetic Immuno Staining Tray, Cell Path, UK). To reduce the endogenous peroxidase, a peroxidase-blocking agent (DAKO S2023) was added for 8 min. Between the different steps, rinsing with buffered saline solution (DAKO S3006) was performed. The sections were incubated for 60 min at room temperature with the rabbit antibody PRSS2 (Center) (Sigma-Aldrich, SAB 1307060) diluted 1:25 in antibody diluent with background reducing components (DAKO S 3022). A secondary antibody (HRP EnVision rabbit (DAKO K4003) was added for 30 min at room temperature. For visualization, DAB (DAKO K3468) was used as chromogen and the slides were counterstained with hematoxylin (DAKO S3301). Multiorgan TMA sections were included as positive and negative controls. The negative controls were obtained by adding antibody diluent without the primary antibody.

### CD8 and FoxP3 immunostaining

Tissue microarray (TMA) sections from formalin-fixed and paraffin-embedded tissue were deparaffinized with xylene, rehydrated in decreasing concentrations of alcohol, and rinsed in distilled water. The slides were boiled in buffered solution (CD8 and TSP-1 at pH 9 [DAKO S2367] and FoxP3 at pH 6 [DAKO S1699]) using a microwave oven for 20 min at 350 W. After cooling and the addition of distilled water to reduce the fluid to room temperature, the slides were moved to a humidifying chamber (Magnetic Immuno Staining Tray, Cell Path, UK). To reduce the endogenous peroxidase, a peroxidase-blocking agent (DAKO S2023) was added for 8 min. Between the different steps, rinsing with buffered saline solution was performed. The sections were incubated at room temperature with the antibody CD8 (DAKO, M7103) diluted 1:100 in antibody diluent with background reducing components (30 min) and with the antibody FoxP3 (Pharmingen, 259D/C7, 560044) diluted 1:25 (60 min). For CD8 and FoxP3, a secondary antibody (HRP EnVision mouse) was added for 30 min at room temperature. For visualization, DAB was used as chromogen and the slides were counterstained with hematoxylin. Multiorgan TMA sections were included as positive and negative controls. The negative controls were obtained by adding antibody diluent without the primary antibody.

### Evaluation of PRSS2 staining in tumor cells

The staining was recorded by a semi-quantitative and subjective grading system. A staining index (SI, values 0–9) was obtained as the product of the staining intensity (none = 0, weak = 1, moderate = 2 and strong = 3) and the proportion of positive tumor cells (0% = 0, 1–10% = 1, 11–50% = 2, >50% = 3). PRSS2 was mainly localized in the tumor cell cytoplasm. Both cytoplasmic and nuclear expression were recorded. Membranous staining was observed in a few cases. In addition to staining benign and malignant prostate tissue, PRSS2 staining was noted in inflammatory cells, nerve tissue and vessels. Results on cytoplasmic expression is reported in this study, and cytoplasmic PRSS2 expression was considered strong for SI ≥ 6 (median in Series 1 and upper quartile in Series 5 [CRPC]).

Intra-observer agreement was tested by blinded re-evaluation of a randomly selected subset of 50 cases from Series 1 and of the 33 cases in Series 5 after two months. Intra-observer agreement was very good or perfect with Kappa values of 0.80 (by SI) and 0.86 (by median SI) for Series 1 and 0.92 (by SI) and 1.0 (by upper quartile SI) for Series 5.

### Evaluation of CD8 and FoxP3

CD8 staining was detected in the cytoplasm and cell membranes, whereas FoxP3 staining was detected in the nuclei. For each staining, one area with the highest number of positive cells was selected (“hot spot”). In these hot spots, one area was counted in high power field (HPF, x400) using an eye-piece graticule (10 × 10 gridlines; 0.25 × 0.25 mm; total 0.0625 mm^2^ for one area. For CD8 and FoxP3, the lower quartile was used as cutoff.

### Statistical analysis

The SPSS statistical package (IBM Corp., Armonk, NY, USA), version 25.0 was used for statistical analyses. Associations between categorical variables were evaluated by Pearson’s chi-square test or Fisher’s exact test. The Mann–Whitney *U* or Kruskal–Wallis tests were used for continuous variables. Univariate survival analyses were carried out by the product-limit method and log-rank tests, Kaplan–Meier plots were made for visualization. Multivariate survival analyses were performed using the Cox´ proportional hazards method and the likelihood ratio test (*P* ≤ 0.15 in univariate survival analyses). We used log–log plots to check for model assumptions of proportionality. Intra-observer agreement was evaluated by Cohen’s kappa.

### Reporting summary

Further information on research design is available in the Nature Portfolio Reporting Summary linked to this article.

## Supplementary information

Supplementary Information

Reporting Summary

Supplementary Table 2

## Data Availability

Clinical data for the patients included in this study are not publicly available per policy to protect patient privacy. Clinical data access, including deidentified individual patient characteristics and survival outcomes can be made available for qualified researchers on a request that does not include revelation of identifiable patient information, upon completion of a Data Transfer Agreement and confirmation of ethical approval. Requests or queries should be directed to the corresponding author. Queries for data access will be answered within a time frame required to ensure high-quality assessment and coordination of the proposed collaborative work. Source data are provided with this paper.

## Source data

Source Data
