# Supplementary material for: PRSS2 remodels the tumor microenvironment via repression of Tsp1 to stimulate tumor growth and progression
Source: Nat Commun. 2022 Dec 27;13:7959. doi: 10.1038/s41467-022-35649-9 (PMC9794699; doi:10.1038/s41467-022-35649-9)
Supplement: Supplementary file 1 — Supplementary Information [file 41467_2022_35649_MOESM1_ESM.pdf]

Supplementary Figures.

Supplementary Figure S1.

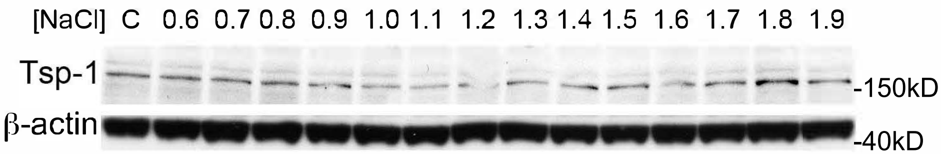

Western blot of WI38 fibroblasts treated with fractions of PC3M-LN4 conditioned media eluted from a heparin-sepharose+Cu^{2+} column with a linear gradient of NaCl plus 20mM imidazole (n=3).

Supplementary Figure S2.

List of proteins present in Tsp-1 repressing fractions (1.0 and 1.1M) and inactive adjacent fractions

0.9M NaCl

~~Keratin 9~~
~~Keratin 1~~
Keratin, CK2
~~Keratin, CK 10~~
Keratin 10
Keratin, CK6a
Keratin, CK14
Keratin, CK6e
Keratin, CK5
Keratin, CK16
Cytokeratin type II
Hornerin
GAPDH
Histone H2A.m
Keratin, CK15
49 kDa protein
Histone H2B.q
ALB protein
Keratin K6irs
Lactotransferrin
~~precursor~~
Histone H4
~~Serotransferrin~~
~~precursor~~
keratin 25 irs1
Hypothetical protein
DKFZp686J1375
Hypothetical protein
DKFZp686B2031
ZF 263
~~MAP3K12~~
Ciliary rootlet
coiled-coil, rootletin
XA protein
Splice Isoform
HMW of Kininogen-
1 precursor
Keratin, CK18
Keratin, Ha6
Cadherin protein
Tigger transposable
element derived 7
Hypothetical protein
FLJ90556

1.0M NaCl

~~Keratin, CK1~~
~~Keratin 9~~
~~Keratin, CK10~~
~~Keratin 2a~~
~~Lactotransferrin~~
~~precursor~~
~~Keratin 1B~~
~~Serotransferrin~~
~~precursor~~
~~ALB protein~~
~~similar to KIAA1501~~
~~24-~~
~~dehydrocholesterol~~
~~reductase precursor~~
~~Tropomodulin 1~~
Protease serine 2
isoform B
~~Hypothetical protein~~
~~FLJ90556~~
~~Splice Isoform 2 of~~
~~WD-repeat protein~~
~~22~~
~~Ciliary rootlet~~
~~coiled-coil, rootletin~~
~~EVH1 domain~~
~~binding protein~~
~~MAP3K12~~
~~Hypothetical protein~~
~~DKFZp434H152~~

1.1M NaCl

~~Keratin 9~~
~~Keratin 1~~
~~Keratin, CK10~~
~~Keratin, CK 2~~
~~Keratin, CK16~~
~~Keratin, CK6C~~
~~Keratin, CK14~~
~~Keratin, CK5~~
~~GAPDH~~
~~Keratin, CK13~~
~~Histone H2A.m~~
~~Histone H2B.q~~
~~Lactotransferrin~~
~~precursor~~
~~Keratin, Hb4~~
~~Serotransferrin~~
~~precursor~~
~~Histone 1, H2aa~~
~~Hypothetical protein~~
~~LOC65250~~
~~DKFZp686J1375~~
~~Desmoglein-1~~
~~Cadherin protein~~
~~similar to KIAA1501~~
~~MAP3K 12~~
~~Hypothetical protein~~
~~DKFZp686B2031~~
~~ALB protein~~
Protease serine 2
isoform B
~~Junction~~
~~plakoglobin~~
~~similar to TP-beta~~
~~similar to CK18~~
~~PRO0650~~
~~Nuclear pore~~
~~membrane~~
~~glycoprotein 210-~~
~~like~~

1.2M NaCl

~~Keratin 1~~
~~Keratin 9~~
~~Keratin, CK10~~
57 kDa protein
Keratin 10
Keratin, CK2
Keratin 1B
Keratin 6L
Keratin, type I
cytoskeletal 14
Keratin 5c
Keratin, CK3
GAPDH
ALB protein
Hypothetical protein
FLJ20261
similar to KIAA1501
similar to KRT8
keratin 25 irs1
ROK1
Cadherin protein
26 kDa protein
hypothetical protein
ABC A13
Tropomodulin 1

Supplementary Figure S3.

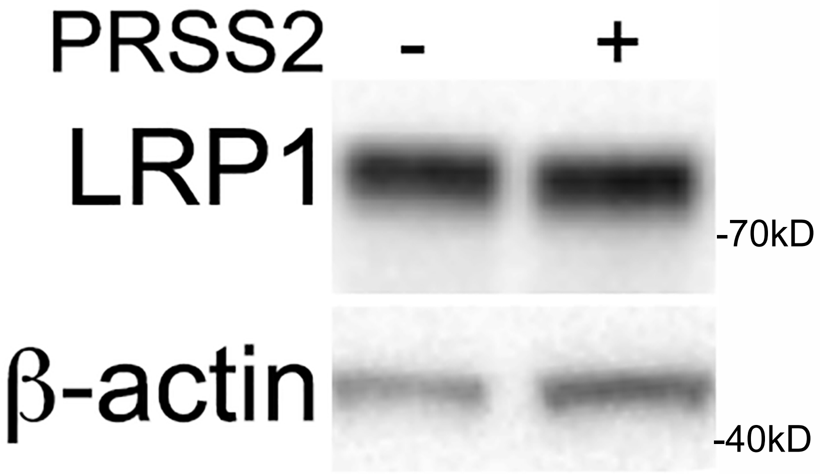

Western blot analysis of Tsp-1, LRP1, and actin expression in MRC5 fibroblasts that were treated with 293T conditioned media alone (-) or conditioned media from 293T cells transfected with pCMV-SPORT6-PRSS2 (n=3).

Supplementary Figure S4.

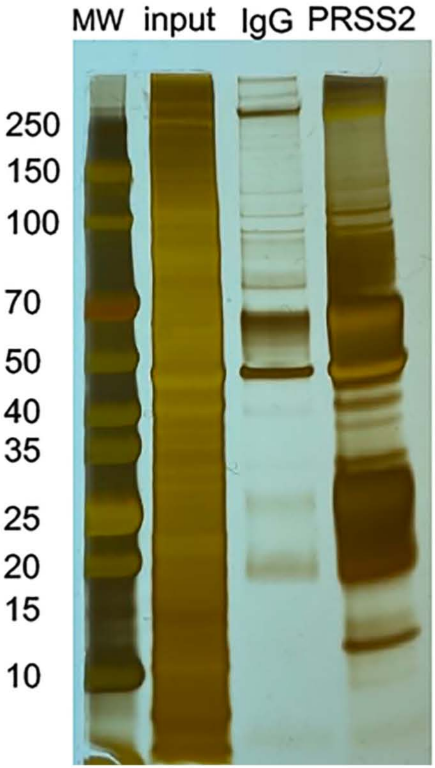

Silver stained polyacrylamide gel loaded with (from left to right) molecular weight markers (MW), input from IP experiment, immunoprecipitated from IgG control (IgG), and immunoprecipitated from α-PRSS2 antibody (PRSS2).

Supplementary Figure S5.
Liist of proteins immunoprecipitated only by PRSS2 that have more than 3 spectral counts

	Spectral Counts		Area Under the Curve
Gene Symbol	PRSS2	IgG	
TRIM21	20	0	1890180000
SVIL	95	0	1596750000
FLNC	70	0	1306770000
STRN3	38	0	1173520000
PLEC	112	0	892116000
MYO18A	26	0	612353000
CTTNBP2NL	39	0	597505000
SPTAN1	64	0	524906000
CORO1C	9	0	478883000
MPRIP	42	0	461029000
TJP1	49	0	431766000
IGKV1D-13	5	0	380172000
STOM	16	0	377938000
IQGAP1	49	0	366736000
FLII	25	0	361198000
TPM1	12	0	332550000
MACF1	117	0	331966000
FLNB	44	0	319585000
CAPZB	12	0	305767000
PDIA6	7	0	302051000
HLA-E	5	0	277995000
UBR5	51	0	248712000
TJP2	33	0	238833000
RAI14	36	0	236049000
ACTN4	33	0	234127000
CLTC	23	0	232240000
DKFZp686J1372	9	0	227785000
LUZP1	39	0	219160000
MYO6	25	0	209570000
RRBP1	19	0	182661000
NES	31	0	179358000
PPP1R18	19	0	172414000
MYO5A	29	0	171120000
MYO1E	17	0	170366000
TMOD3	14	0	163159000
TPM2	4	0	162652000
CTTN	18	0	153889000
LGALS1	5	0	148607000
SPECC1L	31	0	137313000
VDAC2	10	0	132468000
ARPC2	11	0	125070000
MVP	16	0	124451000
A2M	5	0	112823000
STRN4	13	0	111163000
SQOR	11	0	104457000
RPL23	6	0	103869000
ARPC1B	6	0	99707900
HNRNPM	21	0	95921400
C4A	6	0	95083900
ACTR2	16	0	92455600
CAV1	3	0	92114000

TPM1	5	0	87281700
DOCK7	20	0	82621200
RPL24	6	0	82406600
LRRFIP2	13	0	77413500
CAPZA2	12	0	63921100
ACTR3	9	0	59103300
ISG15	3	0	57779300
WDR1	6	0	56617800
CALM2	5	0	56010800
SQSTM1	6	0	52663400
DYNC1H1	34	0	50494600
CEP170	19	0	50289800
CAVIN1	16	0	49687100
MYO10	20	0	49180700
SLC25A3	4	0	46887300
VDAC1	7	0	41187500
ASPH	9	0	40456000
ITGB1	8	0	38116400
ABLIM3	9	0	37103600
SLC25A5	5	0	36571500
AFAP1	10	0	36557600
TMOD2	5	0	35883600
P4HA1	7	0	34660500
SPECC1	13	0	32873900
RPS8	5	0	30367900
RPL4	3	0	30306300
SSFA2	22	0	30296100
ARF4	3	0	30046800
HLA-C	6	0	29705000
TRIOBP	6	0	28547500
RPS4X	5	0	27045400
TMPO	19	0	26906000
PPP1R9B	10	0	26658200
VDAC3	7	0	25915800
TGM2	4	0	24920400
STRIP1	7	0	24646100
FN1	15	0	24492200
TUFM	8	0	24353500
SLMAP	13	0	23954900
LASP1	6	0	23527700
DDX3X	8	0	23481400
COLGALT1	7	0	21826400
RPS3	6	0	21716700
DLST	7	0	20524300
RAB3B	4	0	20460600
PPP1R12C	5	0	20170900
COL6A3	18	0	19681200
TFG	9	0	19591300
RPN2	5	0	18811600
DNAJA1	5	0	18778200
COPA	11	0	18611900
EEF2	5	0	18530900
PABPC1	4	0	17596400
EIF4A1	5	0	17024200
PFKP	7	0	16954700
PPP1CA	5	0	16400800

KIF5B	11	0	16181400
RPS6	3	0	14874800
RUVBL2	6	0	14861700
CEMIP	4	0	14598400
SSH1	7	0	14351300
EMD	3	0	14309300
GJA1	7	0	14288400
DST	26	0	14015100
LGALS3BP	7	0	13858700
DNAJB11	4	0	13662600
ABCD3	4	0	13336700
LRRFIP1	6	0	13291900
RPL7	4	0	13260900
NEK9	4	0	13013300
DNAJA2	3	0	12914200
RAB11FIP1	4	0	12061600
IGLC3	3	0	11999800
FLOT2	7	0	11922500
ACOT9	6	0	11745800
DDX17	5	0	11584100
RNF213	11	0	11515000
SH3BP4	6	0	11479900
TMOD1	8	0	11261700
JCAD	11	0	11058500
RPS3A	3	0	11047700
ERLIN1	4	0	11025000
EPB41L2	9	0	10941400
STRN	9	0	10619400
CLIC1	4	0	10613800
ERLIN2	4	0	10555900
GANAB	10	0	10505600
PLOD1	4	0	10267600
MAP1B	15	0	10151200
IGF2BP3	9	0	10147100
RACGAP1	5	0	10029100
XRCC5	4	0	9820490
MYOF	10	0	9712970
RAB13	3	0	9618030
CYB5R3	3	0	9409190
RPL18	5	0	9387850
ITPR3	11	0	9235000
RPS18	3	0	9210520
RRAS2	3	0	9199800
ATP1A1	9	0	9158490
PCBP1	4	0	8973260
KANK2	5	0	8918050
EPRS	7	0	8795920
DARS	6	0	8677180
CKAP4	6	0	8643520
PCBP2	7	0	8564960
RPL13	3	0	8531740
AASS	3	0	8401660
DDOST	4	0	8388800
F5	3	0	8369430
RUVBL1	3	0	8290570
ATP2B1	6	0	8234260

FLOT1	7	0	8156840
PLOD3	4	0	8048030
B2M	3	0	7942520
DLAT	4	0	7906180
CAD	7	0	7813460
SRGAP2	3	0	7702120
STAT1	6	0	7320940
PABPC4	6	0	7286940
FAM120A	6	0	7232780
GTF2I	7	0	7217940
MICAL2	5	0	7013130
LIMCH1	7	0	6872990
ATP6V1A	7	0	6803790
DDX20	5	0	6592370
LMOD1	4	0	6303040
MX1	4	0	6204520
RAB7A	5	0	6166240
EHD2	3	0	6151180
THY1	3	0	6104270
RPL5	5	0	5962340
ATP2A2	5	0	5955970
IARS	5	0	5901710
KLC1	6	0	5843160
KIF23	7	0	5812760
HADHB	3	0	5811820
COPB2	7	0	5771450
FST	4	0	5657020
TGFBI	4	0	5598210
AHNAK2	5	0	5375960
AP2B1	3	0	5358950
RPL10	3	0	5332270
NSF	6	0	5324620
ZC3HAV1	5	0	5284610
CAMK2D	5	0	5158590
TLN1	8	0	5155710
TCIRG1	3	0	5112590
ICAM1	3	0	5099330
MICAL3	6	0	5067530
ADD1	6	0	5031700
MAP7D1	7	0	4925080
CSRP1	3	0	4903760
RPLP0	3	0	4852000
XRCC6	3	0	4817710
PRKDC	6	0	4768880
PDIA4	4	0	4748170
DNAJC13	10	0	4619970
IFIT1	6	0	4453780
DYNC1I2	3	0	4380370
CYR61	3	0	4375140
PSMD3	6	0	4325630
TRPV2	3	0	4316930
ARHGEF17	7	0	4242570
PALLD	5	0	4154950
MSN	3	0	4100780
PDIA3	5	0	4065620
DCTN4	4	0	4051850

PSMC2	4	0	3867790
SIPA1	4	0	3863240
PLOD2	3	0	3771340
ARF3	3	0	3752890
PPP2R1A	3	0	3720030
DLG5	4	0	3713710
LRCH1	3	0	3596760
ITGA2	3	0	3584370
ADD3	5	0	3507660
XIRP1	5	0	3460690
FILIP1L	5	0	3452440
HSD17B4	5	0	3331970
MYO9B	5	0	3259110
MATR3	8	0	3254420
ARHGDIA	3	0	3183600
IMMT	3	0	3180020
RASAL2	8	0	3168500
AIFM1	4	0	3166970
SYNPO	5	0	3133090
ARHGAP21	5	0	3065580
ATAD3A	4	0	3063610
P4HA2	3	0	3042140
PTX3	5	0	3006770
SHCBP1	3	0	2937870
HADHA	5	0	2888940
CTNND1	3	0	2801550
CACNA2D1	7	0	2800170
RFTN1	4	0	2769760
AKAP8L	4	0	2721010
ERP44	4	0	2695210
CTSB	3	0	2660010
GFPT1	6	0	2632190
PAWR	4	0	2599850
UGGT1	4	0	2568850
RNH1	3	0	2566020
SSR1	3	0	2555350
EDIL3	4	0	2518560
TOM1	4	0	2484870
AP2A1	3	0	2473910
EHD1	3	0	2447210
PEAK1	6	0	2423890
COPB1	4	0	2391190
ATP6V0A1	3	0	2388180
MLEC	6	0	2386570
STRAP	5	0	2314460
SMTN	5	0	2266670
MX2	3	0	2261420
OGDH	3	0	2212360
U2AF2	3	0	2139340
CANX	3	0	2092510
IGKC	3	0	2087520
ACSL3	3	0	2057170
RAB11FIP5	4	0	2024340
STT3A	3	0	2016430
STRIP2	3	0	1992390
TMEM43	3	0	1946820

RHOG	3	0	1852410
PDLIM7	3	0	1843050
EHD4	3	0	1834590
UTRN	6	0	1767060
ARHGEF11	4	0	1745830
PRRC2C	3	0	1735810
INF2	4	0	1726210
PPFIBP1	3	0	1726090
CALU	3	0	1658870
RAB34	3	0	1658440
CAPN2	5	0	1644620
TOM1L2	4	0	1616700
PRRC2A	4	0	1512690
CPT1A	3	0	1511920
WARS	4	0	1501560
MAP4K4	3	0	1478240
SLC38A2	4	0	1458710
CCT6A	3	0	1410580
LRP1	3	0	1365810
RARS	3	0	1359700
OSBPL3	6	0	1343330
SLC25A1	4	0	1336370
MAP4	3	0	1314150
DKK3	5	0	1269030
IGF2BP2	6	0	1245730
CSDE1	3	0	1217850
MAP1A	3	0	1206720
THBS1	3	0	1187540
GAS2L1	4	0	1119960
ENG	3	0	1056210
UGGT2	3	0	1049850
SND1	3	0	1006040
HNRNPL	4	0	927958
MYCBP2	5	0	912300
IFIT3	4	0	838077
DHX9	3	0	812430
ILF3	3	0	753033
SYNPO2	3	0	741934
ETFA	3	0	704462
LAMC1	4	0	660796
SYNCRIP	3	0	652562
GLS	3	0	629496
FASN	4	0	627206
UBA1	3	0	604135
OSBPL8	3	0	456749
ARL1	3	0	407510
APPL2	3	0	401947
ABCC1	3	0	370126
SPAG9	3	0	286035
TKT	3	0	227483

Supplementary Figure S6.

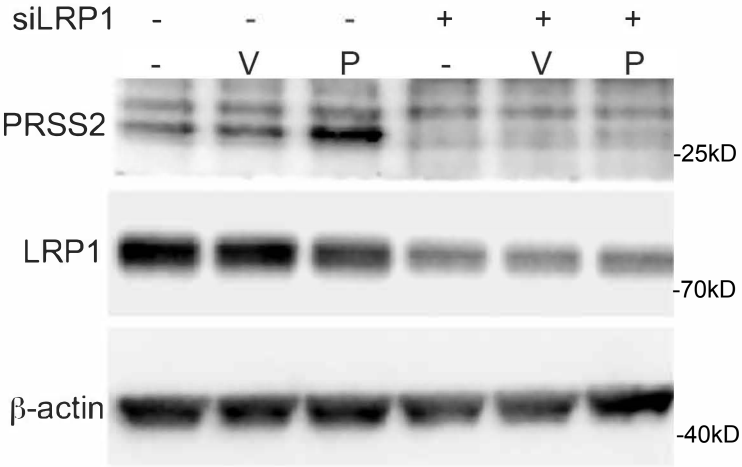

PRSS2 is taken up by cells in an LRP1-dependent manner
Western blot of PRSS2, LRP1, and actin in wild-type and siLRP1 transfected WI38 cells that were untreated (-) or treated with conditioned media from 293T cell transfected with empty pCMV-SPORT6 vector (V) or with pCMV-SPORT6-PRSS2 (P) (n=3).

Supplementary Figure S7.

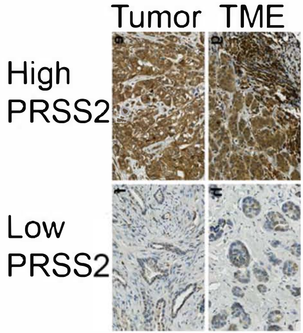

PRSS2 expression in tumor and TME of breast cancer series 2 (n=202 patients).

Supplementary Figure S8.

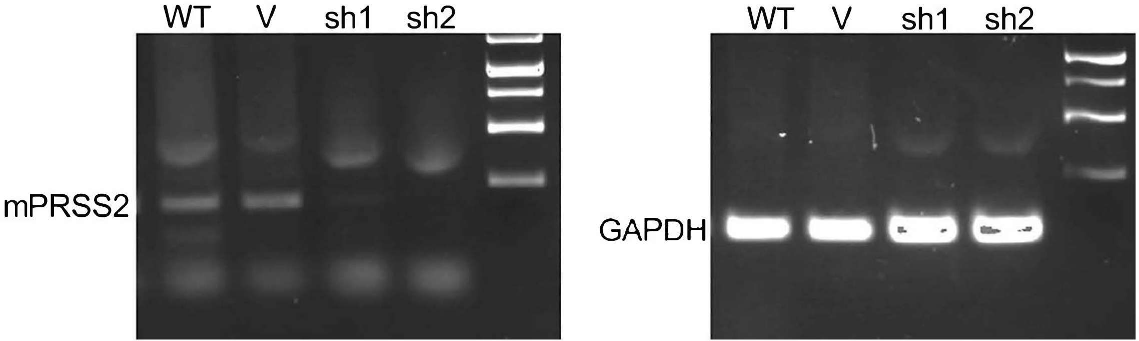

Ethydium bromide stained agarose gels loaded with (left) RT-PCR reactions for PRSS2 from WT, vector control (V), and two independent shPRSS2 transduced (sh1 and sh2) and (right) RT-PCR reactions for GAPDH from WT, vector control (V), and two independent shPRSS2 transduced (sh1 and sh2) (n=3).

Supplementary Figure S9.

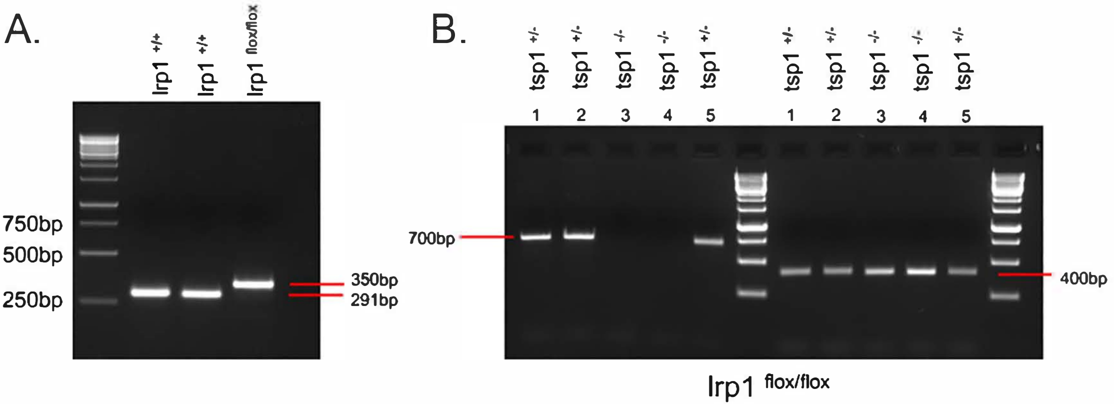

Ethydium bromide stained agarose gels of PCR products from genotyping experiments to confirm:
(A) Myeloid specific knockout of LRP1 as indicated by the presence of the 350bp product and absence of the 291bp product (n=3).
(B) Knockout of Tsp-1 in 5 mice as indicated by the presence of the 400bp band and the absence of the 700bp band as observed in mice #3 and #4 (n=3).

Supplementary Figure S10.

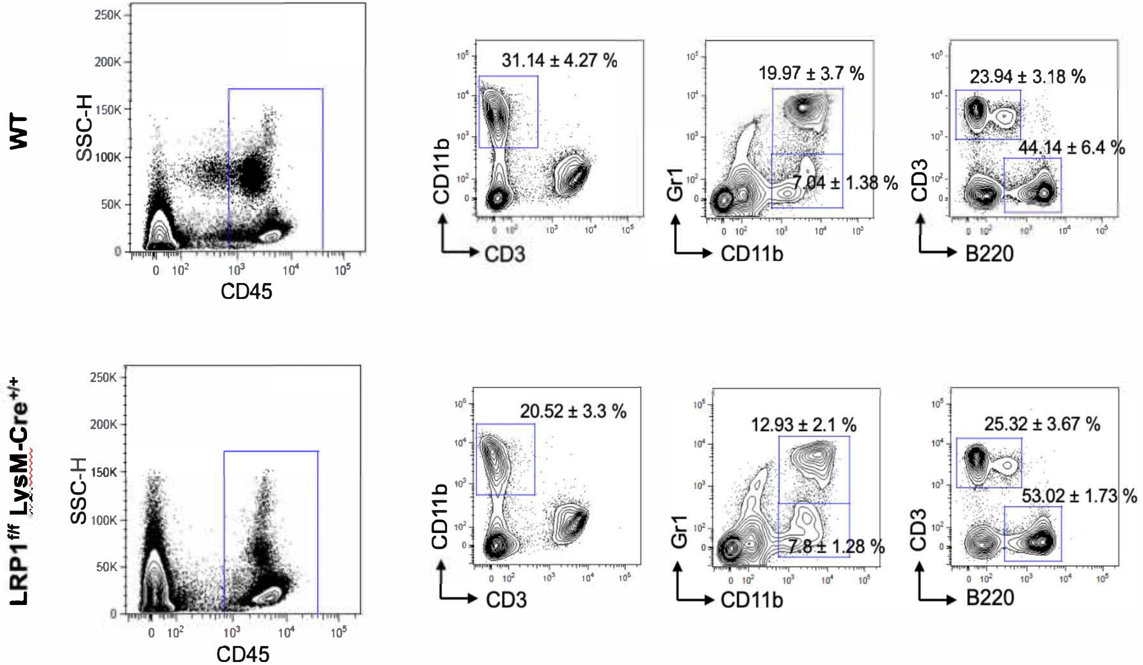

FACS analysis of myeloid and lymphoid cell in WT and LysM-Cre-LRP1$^{f/f}$ mice (n=5 per group).

Supplementary Figure S11.

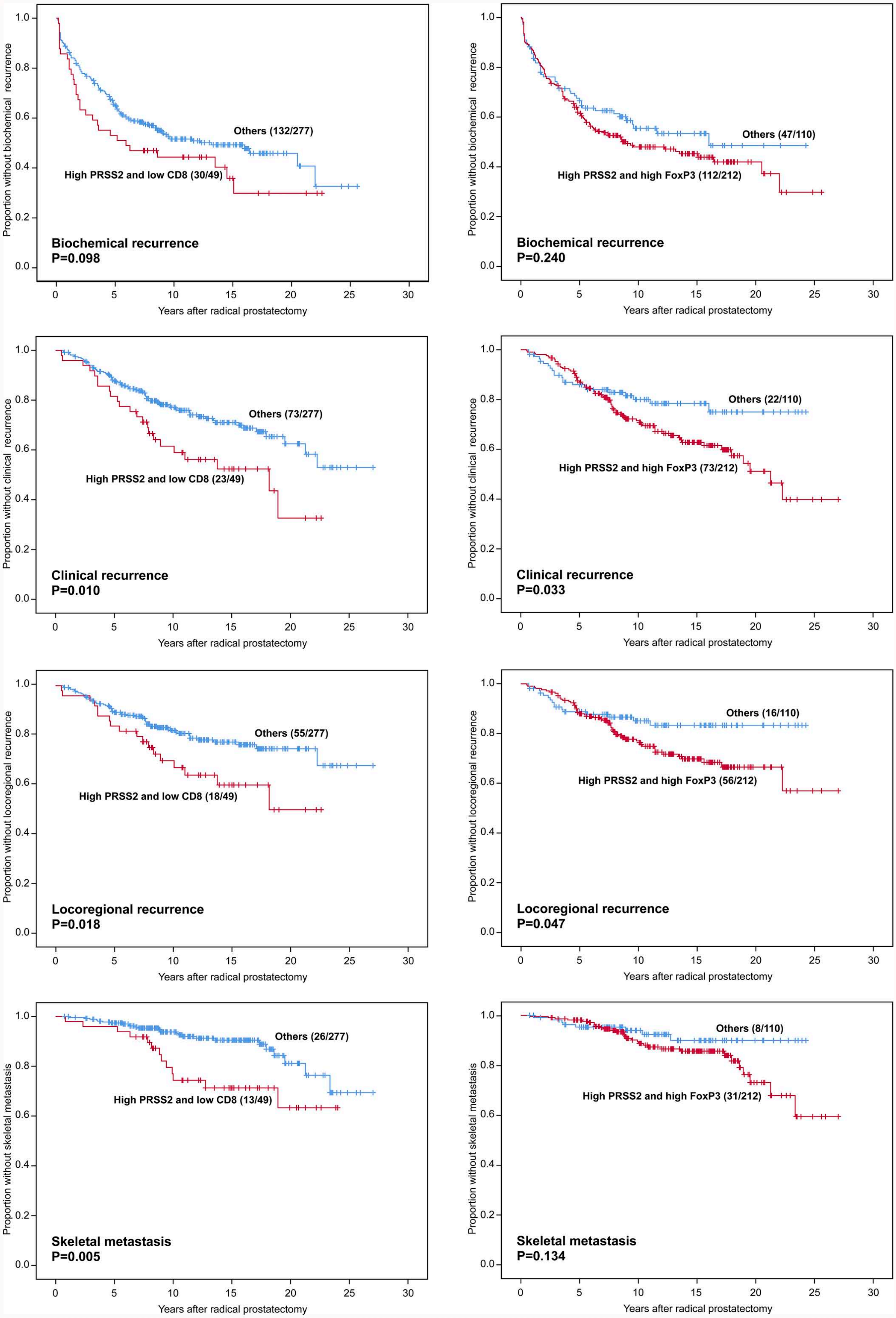

Kaplan-Meier curves of time to biochemical recurrence, clinical recurrence, locoregional recurrence and skeletal metastasis after radical prostatectomy of prostate cancer patients, according to PRSS2 expression and levels of CD8 and FoxP3 T cells.

Supplementary Figure S12.

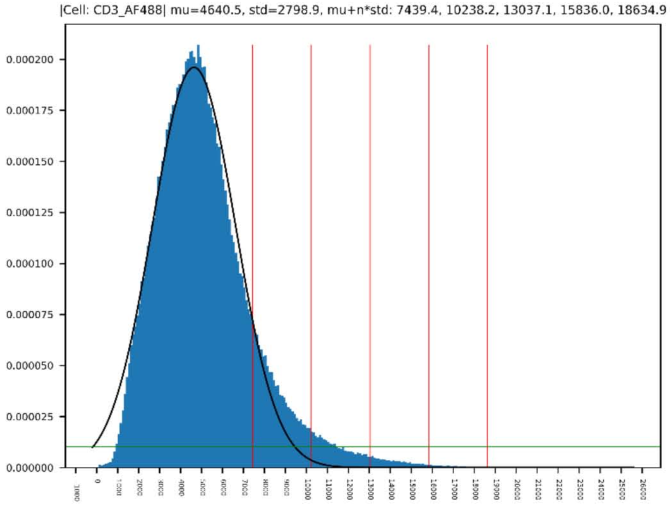

Example of signal analysis to estimate thresholding. The black line is the Gaussian fit estimating its mean and standard deviation values that are listed on top of the image together threshold values (*red vertical lines*) 1, 2, 3, 4, and 5 standard deviation away from the background signal (Gaussian-like peak).

Supplementary Figure S13.

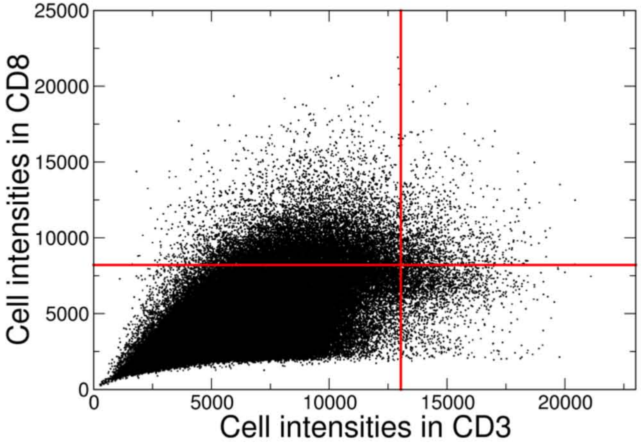

Example of CD3 and CD8 cell intensity correlation with corresponding threshold values (red lines) that are three sigmas away from background. Double-positive cells (CD3+CD8+) are located in the upper right section.

Supplementary Table 1. Associations between PRSS2 expression and selected features in breast cancer (series 2).

Variables	PRSS2 EPITHELIUM					PRSS2 STROMA				
	Low (n=114) n (%)	High (n=58) n (%)	OR	95% CI	p-value[a]	Low (n=130) n (%)	High (n=42) n (%)	OR	95% CI	p-value[a]
**Histologic grade[b]**					<0.001					<0.001
Grade 1-2	66 (78.6)	18 (21.4)	1.0			74 (88.1)	10 (11.9)	1.0		
Grade 3	37 (48.1)	40 (51.9)	3.96	2.00, 7.88		48 (62.3)	29 (32.7)	4.47	2.00, 10.00	
**ER**					0.516					0.006
Pos (≥10%)	61 (68.5)	28 (31.5)	1.0			75 (84.3)	14(15.7)	1.0		
Neg (<10%)	53 (63.9)	30 (36.1)	1.23	0.66, 2.32		55 (66.3)	28 (33.7)	2.73	1.32, 5.66	
**Mitotic count[b]**					0.001					0.004
Low, ≤12.2	85 (71.4)	34 (28.6)	1.0			97 (81.5)	22 (18.5)	1.0		
High, >12.2	18 (42.9)	24 (57.1)	3.33	1.61, 6.91		25 (59.5)	17 (40.5)	3.00	1.39, 6.48	
**CK5/6[c]**					0.439					0.305
Neg, score=0	90 (68.2)	42 (31.8)	1.0			102 (77.3)	30 (22.7)	1.0		
Pos, score>0	24 (61.5)	15 (38.5)	1.34	0.64, 2.81		27 (69.2)	12 (30.8)	1.51	0.68, 3.34	
**p53**					0.029					0.002
Low, score ≤3	87 (71.3)	35 (28.7)	1.0			100 (82.0)	22 (18.0)	1.0		
High, score >3	27 (54.0)	23 (46.0)	2.12	1.07, 4.18		30 (60.0)	20 (40.0)	3.03	1.46, 6.29	
**pMVD[d]**					0.721					0.178
Low (< 1.45)	86 (67.2)	42 (32.8)	1.0			99 (77.3)	29 (22.7)	1.0		
High (≥ 1.45)	25 (64.1)	14 (35.9)	1.15	0.54, 2.43		26 (66.7)	13 (33.3)	1.71	0.78, 3.74	
***BRCA1* mutation**					0.124					0.359
Absent	89 (69.5)	39 (30.5)	1.0			99 (77.3)	29 (22.7)	1.0		
Present	25 (56.8)	19 (43.2)	1.73	0.86, 3.51		31 (70.5)	13 (29.5)	1.43	0.66, 3.09	

Series 2 (n=202). n: number of patients; OR: odds ratio; CI: confidence interval; ER: estrogen receptor; CK5/6: cytokeratin 5/6; pMVD: proliferative microvessel density.

[a] Pearson's chi-squared test.

[b] Eleven cases lack information on histologic grade and mitotic count (mitoses/mm2).

[c] One case lacks information on CK5/6 status.

[d] Five cases lack information on pMVD (FactorVIII+/Ki67+ vessels).
