# Supplementary material for: PRSS2 remodels the tumor microenvironment via repression of Tsp1 to stimulate tumor growth and progression
Source: Nat Commun. 2022 Dec 27;13:7959. doi: 10.1038/s41467-022-35649-9 (PMC9794699; doi:10.1038/s41467-022-35649-9)
Supplement: Supplementary file 2 — Reporting Summary [file 41467_2022_35649_MOESM2_ESM.pdf]

nature portfolio

Corresponding author(s): Randolph S. Watnick

Last updated by author(s): Nov 29, 2022

Reporting Summary

Nature Portfolio wishes to improve the reproducibility of the work that we publish. This form provides structure for consistency and transparency in reporting. For further information on Nature Portfolio policies, see our Editorial Policies and the Editorial Policy Checklist.

Statistics

For all statistical analyses, confirm that the following items are present in the figure legend, table legend, main text, or Methods section.

n/a	Confirmed	
☐	☒	The exact sample size (*n*) for each experimental group/condition, given as a discrete number and unit of measurement
☐	☒	A statement on whether measurements were taken from distinct samples or whether the same sample was measured repeatedly
☐	☒	The statistical test(s) used AND whether they are one- or two-sided<br>*Only common tests should be described solely by name; describe more complex techniques in the Methods section.*
☐	☒	A description of all covariates tested
☐	☒	A description of any assumptions or corrections, such as tests of normality and adjustment for multiple comparisons
☐	☒	A full description of the statistical parameters including central tendency (e.g. means) or other basic estimates (e.g. regression coefficient) AND variation (e.g. standard deviation) or associated estimates of uncertainty (e.g. confidence intervals)
☐	☒	For null hypothesis testing, the test statistic (e.g. *F*, *t*, *r*) with confidence intervals, effect sizes, degrees of freedom and *P* value noted<br>*Give P values as exact values whenever suitable.*
☒	☐	For Bayesian analysis, information on the choice of priors and Markov chain Monte Carlo settings
☒	☐	For hierarchical and complex designs, identification of the appropriate level for tests and full reporting of outcomes
☒	☐	Estimates of effect sizes (e.g. Cohen's *d*, Pearson's *r*), indicating how they were calculated

Our web collection on statistics for biologists contains articles on many of the points above.

Software and code

Policy information about availability of computer code

Data collection	QuPath was used to collect IHC data from whole slides and FACSDiva software was used to obtain FACS data.
Data analysis	FloJo was used to analyze FACS data.

For manuscripts utilizing custom algorithms or software that are central to the research but not yet described in published literature, software must be made available to editors and reviewers. We strongly encourage code deposition in a community repository (e.g. GitHub). See the Nature Portfolio guidelines for submitting code & software for further information.

Data

Policy information about availability of data

All manuscripts must include a data availability statement. This statement should provide the following information, where applicable:

- Accession codes, unique identifiers, or web links for publicly available datasets
- A description of any restrictions on data availability
- For clinical datasets or third party data, please ensure that the statement adheres to our policy

The raw animal and ex vivo source data generated in this study have been submitted and are available as source data. Clinical data for the patients included in this study are not publicly available per policy to protect patient privacy. Clinical data access including deidentified individual patient characteristics and survival outcomes can be made available for qualified researchers on a request that does not include revelation of identifiable patient information, upon completion of a Data Transfer Agreement and confirmation of ethical approval. Requests or queries should be directed to the corresponding

author. Queries for data access will be answered within a time frame required to ensure high quality assessment and coordination of the proposed collaborative work.

Human research participants

Policy information about studies involving human research participants and Sex and Gender in Research.

Reporting on sex and gender	The breast cancer series was comprised entirely of female patients. The prostate cancer series was comprised entirely of males patients.
Population characteristics	This is not applicable
Recruitment	This study involved the analysis of collected tissue from breast and prostate cancer patients and was not a prospective study that involved recruitment.
Ethics oversight	The use of patient data was in accordance with the IRB for the University of Bergen, NO.

Note that full information on the approval of the study protocol must also be provided in the manuscript.

Field-specific reporting

Please select the one below that is the best fit for your research. If you are not sure, read the appropriate sections before making your selection.

☒ Life sciences ☐ Behavioural & social sciences ☐ Ecological, evolutionary & environmental sciences

For a reference copy of the document with all sections, see nature.com/documents/nr-reporting-summary-flat.pdf

Life sciences study design

All studies must disclose on these points even when the disclosure is negative.

Sample size	All animal experiments are designed with cohorts of n=8 to achieve a 95% confidence based on Power Analysis with an of 0.05 and an f2 of 0.35.
Data exclusions	Data was excluded for animals that died due to causes unrelated to the experimental aims.
Replication	All in vitro experiments were performed with a minimum of 3 independent biological replicates. All attempts to replicate experimental results were successful.
Randomization	Randomization was not required as no treatments were performed on animals.
Blinding	The pathologists who analyzed the human and mouse IHC staining were blinded to the identify of the subjects..

Reporting for specific materials, systems and methods

We require information from authors about some types of materials, experimental systems and methods used in many studies. Here, indicate whether each material, system or method listed is relevant to your study. If you are not sure if a list item applies to your research, read the appropriate section before selecting a response.

Materials & experimental systems

n/a	Involved in the study	
☐	☒	Antibodies
☐	☒	Eukaryotic cell lines
☒	☐	Palaeontology and archaeology
☐	☒	Animals and other organisms
☐	☒	Clinical data
☒	☐	Dual use research of concern

Methods

n/a	Involved in the study	
☒	☐	ChIP-seq
☐	☒	Flow cytometry
☒	☐	MRI-based neuroimaging

Antibodies

Antibodies used	Thrombospondin 1 Abcam Cat# ab263905<br>LRP1 Cell Signaling Technology Cat#64099

pJNK (Thr183/Tyr185) Cell Signaling Technology Cat#4668
JNK Cell Signaling Technology Cat#9252
c-Jun Cell Signaling Technology Cat#9165
Phospho-c-Jun (Ser73) Cell Signaling Technology Cat#3270
c-myc Cell Signaling Technology Cat#9402
ITGA2 Cell Signaling Technology Cat#88228
ITGB1 Cell Signaling Technology Cat#4706
β-actin Abcam Cat# ab6276
beta-actin mouse Abcam Cat# mAb AC-15,
PRSS2 Abcam Cat# ab223064
murine Thrombospondin-1 Abeam Cat# ab226383
murine CD4 Abcam, Cat# ab183685
murine Foxp3 R&D Systems, Cat# MAB8214
murine : CD3 Thermo Fisher Scientific, Cat# PAl-29547
murine CD8 Cell Signaling, Cat# 98941
human PRSS2 Sigma-Aldrich, Cat# SAB 1307060
human CD8 DAKO, Cat# M7103
human FoxP3 (harmingen, Cat# 259D/C7, 560044

Validation

All primary antibodies used were commercially validated by the supplier.

Eukaryotic cell lines

Policy information about cell lines and Sex and Gender in Research

Cell line source(s)

E0771 cells were purchased from ATCC
SUM159 cells were derived by Stephen Ethier when he was at the University of Michigan, we received them from him as a gift after he had moved to the University of South Carolina Medical College.
Pan02 cells are from NCI DCTD TUMOR REPOSITORY A CATALOG OF IN VITRO CELL LINES, TRANSPLANTABLE ANIMAL AND HUMAN TUMORS, CANINE SPECIMENS AND YEAST Biological Testing Branch Developmental Therapeutics Program Division of Cancer Treatment and Diagnosis National Cancer Institute National Institutes of Health DCTD
Wl-38 cells were a gift of the Weinberg Lab (MIT) they were originally purchased from ATCC.
Primary human peripheral blood mononuclear cells were purchased from Lonza

Authentication

Cells obtained from ATCC were not authenticated. Wl-38 cells were authenticated by the Weinberg lab prior to transfer, we confirmed identify based on the expression of LRPl as published. SUM159 cells were derived by Stephen Ethier and confirmed to us at the time we obtained them. Pan02 cells were not authenticated as they originated from the NCI.

Mycoplasma contamination

All cell lines except primary human peripheral blood mononuclear cells were tested for mycoplasma contamination by PCR testing, none tested positive.

Commonly misidentified lines
(See ICLAC register)

No commonly misidentified cell lines were used.

Animals and other research organisms

Policy information about studies involving animals; ARRIVE guidelines recommended for reporting animal research, and Sex and Gender in Research

Laboratory animals

Female SCID mice (6-8 weeks old) were purchased from Massachusetts General Hospital.
Myeloid-specific LRP1 knockout mice were generated by crossing LysM-Cre mice with LRP1flox/flox mice.
LRP1 and Tsp-1 double knock out mice (DKO) were generated by crossing LysM-Cre-LRP1-/- mice with Tsp-1-/- mice. The double knockout was confirmed by genotyping.
C57bl6/J mice were purchased from Jackson Laboratory.

Wild animals

No wild animals were used for this study.

Reporting on sex

For breast cancer models only female mice were used.
For pancreatic cancer models male and female mice were used at equal proportions.

Field-collected samples

No field collected samples were used for this study.

Ethics oversight

All animal work was conducted in accordance with a protocol approved by the Boston Children's Hospital Animal Care and Use Committee.

Note that full information on the approval of the study protocol must also be provided in the manuscript.

Clinical data

Policy information about clinical studies

All manuscripts should comply with the ICMJEguidelines for publication of clinical research and a completedCONSORT checklist must be included with all submissions.

Clinical trial registration	N/A
Study protocol	The study was approved by the Western Regional Committee for Medical and Health Research Ethics, REC West (REK 2014/1984) (Series 1) and the Institutional Revie Board at McGill University Hospital, A03-M33-02A (Series 2). All studies were performed in accordance with guidelines and regulations by the University of Bergen and REK, and in accordance with the Declaration of the Helsinki Principles.
Data collection	*Describe the settings and locales of data collection, noting the time periods of recruitment and data collection.*
Outcomes	Data were analyzed using SPSS Statistics for Windows, Version 25.0 (IBM Corp, Armonk, NY, USA). Statistical significance was assessed at the two-sided 5% level, whereas borderline statistical significance was defined as P-values between 5 and 10%. Associations between categorical variables were evaluated using Pearson's X^2 test of Fisher's exact test, as appropriate, and odds ratios (OR) were computed. Univariate survival analyses were carried out using the Kaplan-Meier method with significance determined by log-rank test. The endpoint in survival analysis was breast cancer specific survival (BCS) (Series 1). Entry data was the date of diagnosis. Patients who died from other causes were censored at the date of death.

Flow Cytometry

Plots

Confirm that:

- [x] The axis labels state the marker and fluorochrome used (e.g. CD4-FITC).
- [x] The axis scales are clearly visible. Include numbers along axes only for bottom left plot of group (a 'group' is an analysis of identical markers).
- [x] All plots are contour plots with outliers or pseudocolor plots.
- [x] A numerical value for number of cells or percentage (with statistics) is provided.

Methodology

Sample preparation	For analysis of peripheral blood, blood was collected from the tails of mice in anti-coagulant buffer (PBS with 5mM EDTA). Red blood cells were eliminated by incubation with lysis buffer (BD Bioscience) for 10 minutes at RT. Cell suspensions were pre-blocked with 2% FBS plus Fc block (CD16/CD32, 1:30, BD Biosciences Pharmingen) and then incubated with primary antibodies.
Instrument	LSRII flow cytometer BD Biosciences
Software	FACSDiva software (BD Biosciences)
Cell population abundance	Samples were only analyzed for abundance using the FACSDiva software and were not propagated or used subsequent to flow cytometry.
Gating strategy	Flow cytometry analysis was performed using a variety of controls including isotype antibodies, fluorescence minus one (FMC) samples, and unstained samples for determining appropriate gates, voltages, and compensations required in multivariate flow cytometry. For sorting, targeted cell populations were gated within FACSDiva software and sorted by Aria II sorter (BD Biosciences).

- [x] Tick this box to confirm that a figure exemplifying the gating strategy is provided in the Supplementary Information.
