# Supplementary material for: PRSS2 remodels the tumor microenvironment via repression of Tsp1 to stimulate tumor growth and progression
Source: Nat Commun. 2022 Dec 27;13:7959. doi: 10.1038/s41467-022-35649-9 (PMC9794699; doi:10.1038/s41467-022-35649-9)
Supplement: Supplementary file 3 — Supplementary Table 2 [file 41467_2022_35649_MOESM3_ESM.pdf]

Supplementary Table 2. Multivariate survival analysis (Cox proportional hazards method) for 338 patients with localized prostatic carcinomas (radical prostatectomies), using biochemical recurrence, clinical recurrence and loco-regional recurrence as end-points.

Variables	No. of patients	HR[1]	95 % CI[2]	P value[3]
**Biochemical recurrence**				
Gleason score[4]				
≤3+4	222	1.0		
≥4+3	106	2.6	1.9-3.7	<0.0005
Pathological stage[5]				
pT2	196	1.0		
≥pT3	132	2.0	1.4-2.8	<0.0005
Preoperative s-PSA[6]				
Low	246	1.0		
High	82	1.8	1.3-2.5	0.001
PRSS2[7]				
Low	93	1.0		
High	235	1.4	1.0-2.1	0.052p
**Clinical recurrence**				
Gleason score[4]				
≤3+4	228	1.0		
≥4+3	106	2.8	1.9-4.3	<0.0005
Pathological stage[5]				
pT2	196	1.0		
≥pT3	138	2.2	1.4-3.4	<0.0005
PRSS2[7]				
Low	93	1.0		
High	241	2.3	1.3-3.9	0.001
**Loco-regional recurrence**				
Gleason score[4]				
≤3+4	228	1.0		
≥4+3	106	2.5	1.6-4.1	<0.0005
Pathological stage[5]				
pT2	196	1.0		
≥pT3	138	2.3	1.4-3.7	0.001
PRSS2[7]				
Low	93	1.0		
High	241	2.5	1.3-4.8	0.002

[1]Hazard ratio.
[2]Confidence interval.
[3]Likelihood ratio test.
[4]Gleason score in radical prostatectomy specimens.
[5]Pathological stage, UICC TNM Classification of malignant tumours, Eighth edition, 2017.
[6]s-PSA, cut-off by upper quartile.
[7]Cytoplasmic staining, cut-off by median.
